# Targeting microRNA-dependent control of X chromosome inactivation improves the Rett Syndrome phenotype

**DOI:** 10.1038/s41467-025-61092-7

**Published:** 2025-07-04

**Authors:** Song Lou, Rachisan DJiake Tihagam, Urszula N. Wasko, Zaffar Equbal, Sanjay Venkatesan, Klaudia Braczyk, Piotr Przanowski, Bon Il Koo, Ilyas Saltani, Arjun Tushir Singh, Shibi Likhite, Samantha Powers, George M. P. R. Souza, Robert A. Maxwell, Jun Yu, Lihua J. Zhu, Mark Beenhakker, Stephen B. G. Abbott, Zhipeng Lu, Michael R. Green, Kathrin C. Meyer, Jogender Tushir-Singh, Sanchita Bhatnagar

**Affiliations:** 1 Department of Medical Microbiology and Immunology, University of California Davis School of Medicine, Davis, CA USA; 2 Department of Biochemistry and Molecular Genetics, University of Virginia School of Medicine, Charlottesville, VA USA; 3 Center for Gene Therapy, Nationwide Children’s Hospital, Columbus, OH USA; 4 Department of Pharmacology, University of Virginia School of Medicine, Charlottesville, VA USA; 5 The Vincent J. Coates Proteomics/Mass Spectrometry Core Laboratory, University of California, Berkeley, CA USA; 6 Department of Molecular, Cell, and Cancer Biology, University of Massachusetts Medical School, Worcester, MA USA; 7 University of Southern California Alfred E. Mann School of Pharmacy and Pharmaceutical Sciences, USC Norris Comprehensive Cancer Center, USC Eli and Edythe Broad CIRM Center for Regenerative Medicine and Stem Cell Research, Los Angeles, CA USA; 8 Department of Pediatrics, The Ohio State University, Columbus, OH USA; 9 The M.I.N.D. Institute, University of California at Davis, Davis, CA USA

**Keywords:** RNA, DNA

## Abstract

X chromosome inactivation (XCI) is induced by *Xist* long non-coding RNA and protein-coding genes. However, the role of small non-coding RNA function in XCI remains unidentified. Our genome-wide, loss-of-function CRISPR/Cas9 screen in female fibroblasts identified microRNAs (miRNAs) as regulators of XCI. A striking finding is the identification of miR106a among the top candidates from the screen. Loss of miR106a is accompanied by altered *Xist* interactome, leading to dissociation and destabilization of *Xist*. XCI interference via miR106a inhibition has therapeutic implications for Rett syndrome (RTT) girls with a defective X-linked *MECP2* gene. Here, we discovered that the inhibition of miR106a significantly improves several facets of RTT pathology: it increases the life span, enhances locomotor activity and exploratory behavior, and diminishes breathing variabilities. Our results suggest that miR106a targeting offers a feasible therapeutic strategy for RTT and other monogenic X-linked neurodevelopmental disorders.

## Abstract (web summary)

X chromosome inactivation (XCI) is crucial for gene regulation, yet the role of small non coding RNAs in this process was unclear. Here, the authors identify miR106a as a key XCI regulator and show its inhibition improves Rett syndrome symptoms in a mouse model.

## Introduction

X chromosome inactivation (XCI) is an epigenetic silencing mechanism that balances X-linked gene expression among XX females and XY males^1^. XCI is a coordinated multistep process that includes X chromosome counting to silence all but one, random choice of the inactive X chromosome (X_i_), and spreading silencing along the X chromosome^2^. Several of these functions are moderated by long non-coding RNAs (lncRNA) enriched in the X chromosome^3^. Of particular interest is *Xist* lncRNA, which covers the X_i_ along the length in *cis* and induces a repressive dynamic heterochromatin state. Mechanistically, *Xist* lncRNA is linked to histone modifications, DNA methylation at CpG islands, nucleosomal remodeling, chromatin compaction, and X_i_ repositioning to the nuclear periphery^4,5^. How these *Xist*-centric-diverse pathways collaborate to initiate and maintain XCI remains to be understood. A comprehensive functional and structural analysis identified highly conserved repetitive motifs at the 5’ region of *Xist*, referred to as repeat A (RepA), critical for silencing the X chromosome^6,7^. Loss of *RepA* is associated with the biallelic expression of X-linked genes and enrichment of active transcriptional marks on the X_i_^6,8^. The binding of regulatory proteins to *RepA* recruits repressive complexes, including NR corepressor, the silencing mediator of retinoic acid or thyroid hormone receptor, and nucleosome remodeling deacetylase complex (reviewed^9^). Another study demonstrated the exclusive binding of Spen, Rnf20, and Wilms’ tumor 1-associating protein (Wtap) to A-repeat in *Xist* using an inducible male ESC cell line^10^. Spen is required for transcriptional repression upon differentiation but does not affect *Xist* accumulation or spreading across X_i_^10^. Rnf20 enhances transcription via ubiquitination of histone H2B^11^. Wtap is required to accumulate the methyltransferase complex (RBM15/15B/METTL3/METTL14) that directs post-transcriptional N^6^-methyladenosine (m^6^A) formation on *Xist*^12^.

Additionally, as shown recently, *trans*-acting X chromosome inactivation factors (XCIF) also regulate the X_i_ silencing. Several proteomic, genetic, and computational screens identified protein-coding gene regulators of XCI^10,13–16^. We have previously identified and characterized XCIFs that target *Xist* transcriptionally^13^. Significantly, the pharmacological or genetic targeting of the XCIFs is sufficient to disrupt the X_i_ silencing ex vivo^13^ and in vivo^17^. We note that for reasons such as incomplete coverage of the libraries, lack of phenotypic changes, and functional redundancy, additional XCIFs could be identified.

microRNA (miRNA)-driven post-transcriptional gene silencing mechanism regulates diverse biological processes, including cell proliferation, embryonic stem cell differentiation, apoptosis, and tissue development^18,19^. miRNAs are small non-coding RNAs, for which target specificity is dictated by the 6–8 nucleotide seed region at the 5’-end (reviewed^20^). miRNAs pair with Argonaute (Ago) proteins to form the cytoplasmic miRNA-induced silencing complex, which alters the transcriptional output by mRNA degradation. Systematic analysis of the subcellular distribution of miRNA and small RNA sequencing determined that mature miRNAs are present both in the nucleus and cytosol^19,21^. While the canonical function of miRNAs in mRNA stability is well characterized, their nuclear function is poorly understood. Nonetheless, several nuclear-localized miRNAs are linked to transcriptional and post-transcriptional gene regulation through promoter activation, transcriptional gene silencing, and alternative splicing^19^. Interestingly, the miRNA abundance in the X chromosome and their predicted interactions with *Xist* suggest miRNA involvement in XCI^22–24^. However, the transcriptional outcomes of miRNA-*Xist* interactions and the molecular function of miRNA in XCI are still largely unknown.

Restoration of a normal gene copy from the X_i_ is a viable therapeutic approach for multiple X-linked, monogenic neurodevelopmental disorders, such as Rett syndrome (RTT)^25^. RTT females carry heterozygous loss-of-function mutations in methyl CpG-binding protein 2 (*MECP2)*, essential for neuronal morphology and synaptic function^26^. Although there is no cure for RTT, activating a quiescent *Mecp2* gene improved several phenotypes in adult RTT mice^27^. Analogously, we previously demonstrated the feasibility and safety of expressing a healthy copy of X_i_-linked MECP2 by genetic and pharmacological targeting of XCIFs in vivo^13,17^. Here, we tested whether miR106a inhibition rescues MECP2 function and improves dysfunctional neuronal phenotypes, motor function, and behavioral deficits in multiple RTT preclinical models.

## Results

### Genome-wide CRISPR-Cas9 screen identifies miRNAs as regulators of mammalian XCI

**Fig. 1:**
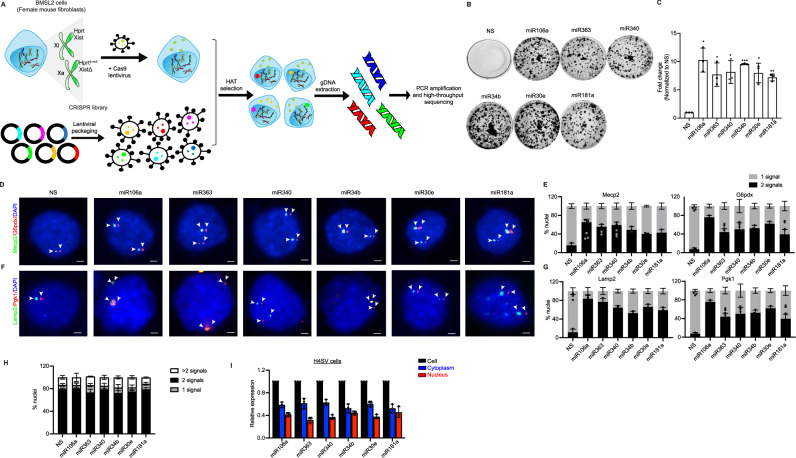
A CRISPR/Cas9 screen identifies miRNAs as regulators of mammalian XCI. **A** Schematic summary of the CRISPR/Cas9 screen in female mouse fibroblast BMSL2 cells. The active X chromosome (X_a_) harbors the deletion of *Xist*. **B, C** BMSL2 cells expressing a sgRNA against the indicated miRNAs or, as a control, a non-silencing (NS) sgRNA were selected in the HAT medium. The representative images are shown (**B**), and the results are quantified from three experiments after crystal violet staining (**C**). Unpaired, two-sided t-test with no multiple adjustments. For miR106a, *p = 0.017; miR363, *p = 0.0303; miR340, *p = 0.026; miR34b, ***p = 4.21 × 10^-5^; miR30e, *p = 0.02; miR181a, **p = 0.0032. **D–G** Two-color RNA FISH monitoring expression of *G6pdx* (*Red*) and *Mecp2* (*Green*), and *Pgk1* (*Red*) and *Lamp2* (*Green*) in each of the six miRNAs knockdown H4SV cell lines. DAPI staining is shown in blue. The representative images are shown (**D, F**), and the results are quantified from three experiments (**E, G**). **H** DNA FISH monitoring X chromosome content in the six miRNA KD H4SV cell lines is quantified from three experiments. **I** qRT-PCR analysis monitoring miRNA levels in the whole-cell, nuclear, and cytoplasmic fractions from H4SV cells. Data were analyzed from three experiments. Data is expressed as mean ± SD (**C, E, G, H, I**). Scale bars: 5 µm (**D, F**).

To globally search for XCIF, we performed a near genome-wide, loss-of-function CRISPR/Cas9 screen in a female mouse embryonic fibroblast cell line [BMSL2^13^], which bears a hypoxanthine-guanine phosphoribosyltransferase (*Hprt*) only on the X_i_. After viral delivery of the sgRNA library, cells persisting in hypoxanthine-aminopterin-thymidine (HAT) selection media were enriched, and sgRNAs were identified (Fig. 1A). We next established a pipeline to prioritize candidates based first on the representation of sgRNA above the threshold and then on the number of sgRNAs in replicates. The candidate protein-coding genes identified were known or predicted to function in metabolic pathways, protein-protein interaction, molecular transducers, signal transduction, developmental pathways, chromatin binding, transporter activity, and X chromosome biology (Supplementary Data I, Supplementary Fig. 1A).

Surprisingly, we also identified miRNAs whose CRISPR-mediated knockdown expressed X_i_-linked *Hprt* (Supplementary Fig. 1B) and enabled survival in the HAT selection medium (Fig. 1B, C). The knockdown of these miRNAs in an unrelated female mouse fibroblast cell line [H4SV^13^] expressed X_i_-linked *GFP* transgene (Supplementary Fig. 1C, D). We note that the inhibition of miR17a, a paralog of miR106a and miR363, did not increase the X_i_-linked genes (Supplementary Fig. 1E). The specificity of each sgRNA was confirmed by monitoring the expression of miRNAs and their previously validated target genes level (Supplementary Fig. 1F, G). Consistently, the synthetic miRNA inhibitors increased *GFP*, *Hprt*, and their corresponding target genes in H4SV cells (Supplementary Fig. 1H, I), ruling out off-target effects.

Next, we analyzed the expression of four X-linked genes, glucose-6-phosphate dehydrogenase X-linked (*G6pdx*), Methyl CpG-binding protein 2 (*Mecp2*), lysosomal-associated membrane protein 2 (*Lamp2*), and phosphoglycerate kinase 1 (*Pgk1*), using two-color RNA fluorescence in situ hybridization (FISH). In H4SV cells expressing a control NS sgRNA, RNA FISH revealed a single nuclear signal for *G6pdx*, *Mecp2, Lamp2*, and *Pgk1*, indicative of monoallelic expression (Fig. 1D–G). In contrast, each miRNA knockdown cell line showed a substantial increase in the fraction of cells with biallelic nuclear signal (Fig.1D–G), which affected the average transcript abundance for *G6pdx* (~1.11-fold), *Mecp2* (~1.31-fold), *Lamp2* (~1.28-fold), and *Pgk1* (~1.24-fold) relative to control (Supplementary Fig. 1J). The X-chromosome content of the miRNA knockdown cell lines was similar to that of the control cell line (Fig. 1H and Supplementary Fig. 1K). The results of these experiments identified six miRNAs as XCI regulators: miR106a-5p (miR106a), miR363-3p (miR363), miR340-5p (miR340), miR34b-5p (miR34b), miR30e-5p (miR30e), and miR181a (Supplementary Fig. 1L). The gene ontology analysis revealed that the six miRNAs were known or predicted to be involved in diverse cellular processes, including apoptosis, interleukin production, transporter activity, and receptor signaling (Supplementary Fig. 1M).

The systematic analysis of each of the miRNAs’ cellular distribution confirmed their presence in nuclear and cytoplasmic fractions (Fig.1I). Fraction purity was confirmed by analyzing nuclear (histone H3) and cytoplasmic (β-tubulin) marker proteins, nuclear-enriched lncRNAs (Malat1 and Neat), and cytoplasmic-enriched transfer RNAs (tRNA-Lys-TTT and tRNA-Met-CAT; Supplementary Fig. 1N–P).

### miR106a physically interacts with *RepA* in *Xist* RNA

**Fig. 2:**
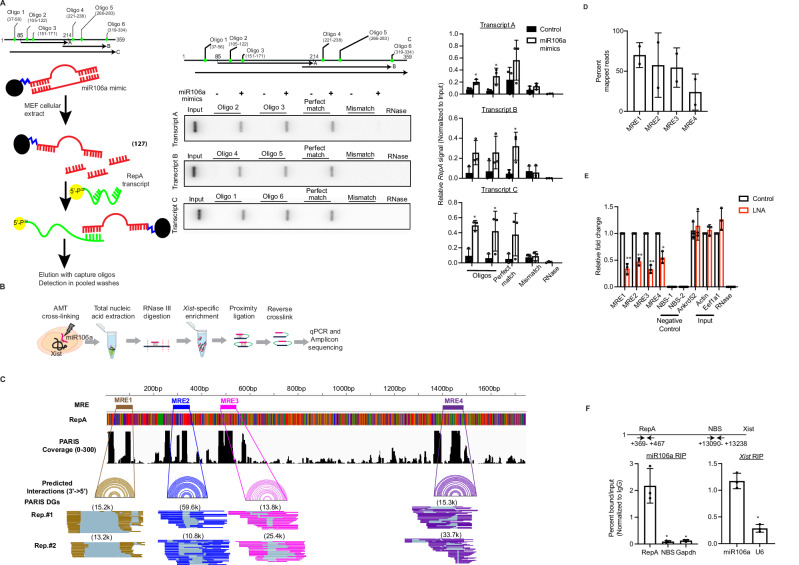
miR106a physically interacts with *RepA* in *Xist* RNA. **A** Strategy to capture miR106a-*RepA* complex (*Left*). Competitive elution of *RepA* from miR106a-RepA complex using mismatch, perfect, or imperfect complementary capture oligonucleotides. Samples pretreated with RNase were used as a negative control. The experiment was performed three times; a representative image from one experiment is shown (*Middle*), and the results are quantified (*Right*) from three different experiments. For transcript A, Oligo 2, *p = 0.013, Oligo 3, *p = 0.034, Perfect match ^ns^*p* = 0.22; For transcript B, Oligo 4, ^ns^*p* = 0.061, Oligo 5, ^ns^*p* = 0.17, Perfect match ^*^p = 0.031; For transcript C, Oligo 1, **p = 0.0034, Oligo 6, ^ns^*p* = 0.083, Perfect match ^ns^*p* = 0.126. **B** Outline of the modified PARIS2 method. **C** PARIS2 assay validates predicted miR106a interactions (Colored arcs) in the *RepA* region. Fractions of duplex groups (DGs) corresponding to each site are shown. Numbers in parentheses are the counts of gapped alignments in each DG. *n* = 2. **D** The percentage of mapped reads for each amplicon from the replicates is shown. Data were analyzed from two experiments and expressed as mea*n* ± SD. **E** qRT-PCR analysis following PARIS2 assay monitoring the enrichment of miR106a-*RepA* duplex in control and LNA-treated cells. The predicted miR106a non-binding region (NBS) in *Xist* is a negative control. *n* = 3. For MRE 1, **p = 0.0072; MRE2, **p = 0.0056; MRE3, **p = 0.0043; MRE4, *p = 0.020. **F** RIP assay monitoring miR106a-*Xist* interaction in H4SV cells using biotinylated miR106a (*Left*) and *Xist*-specific probes (*Right*). The predicted miR106a non-binding region (NBS) in *Xist* and U6 is a negative control. *n* = 3. For miR106sp RIP, NBS, *p = 0.029; Gapdh, *p = 0.03. For Xist RIP, *p = 0.015. Data is expressed as mean ± SD (**A, E, F**). Unpaired, two-sided t-test with no multiple adjustments (**A, E, F**).

qRT-PCR analysis revealed that levels of *Xist*, a critical mediator of XCI, were reduced to varying extents in cells depleted for miR106a, miR34b, and miR30e (Supplementary Fig. 2A), raising the possibility of miRNA-mediated *Xist* regulation. Knockdown of miR363, miR181, and miR340 did not affect *Xist* levels (Supplementary Fig. 2A), indicating *Xist*-independent mechanisms of XCI regulation through indirect or global interactions. The sequence analysis of *Xist* RNA revealed that miRNA recognition elements (MRE) for miR106a, but not miR34b and miR30e, were concentrated in the 5’ region of Xist, defined as *RepA* (Supplementary Fig. 2B). We, therefore, hypothesized that miR106a could modulate *Xist* function in XCI via *RepA*. To test this idea, we analyzed the in vitro binding of miR106a with RepA sub-fragment using an electrophoretic mobility shift assay. The miR106a shifted *RepA* fragment, harboring miR106a MRE in a concentration-dependent manner, with over 60% miR106a binding at ~60 nM concentration (Supplementary Fig. 2C). On the other hand, miR106a poorly bound the *RepA* with mutated miR106a MRE and tRNA (Supplementary Fig. 2C). Consistently, the competitive elution of RNA species in complex with miR106a enriched for *RepA* fragment following elution with capture oligonucleotides, but not with the non-interacting and mismatched oligonucleotides (Fig. 2A). RNase treatment of *RepA* transcript prior to miR106a abolished *RepA* signal (Fig. 2A). We next adapted a conformation-dependent fluorescent RNA sensor to encode a miR106a-specific MRE in *RepA* (Supplementary Fig. 2D, *Left*). Strikingly, the fluorescence intensity of bound DFHBI fluorophore increased ∼10-fold for RNA sensor harboring miR106a but not mutated miR106a MRE (Supplementary Fig. 2D, *Right*). Finally, the miR106a accumulates with the *RepA*-positive nuclear region in NIH3T3 cells ectopically expressing *RepA* fragment (Supplementary Fig. 2E), indicating miR106a-*RepA* pairing.

Prompted by these findings, we interrogated the miR106a occupancy in endogenous *Xist* in a series of experiments. As a first step, we directly evaluated the miR106a-*Xist* interactions in a living cell through a modified psoralen analysis of RNA interactions and structures (PARIS2^28^; Fig. 2B). The sequence analysis and computational modeling of PARIS2-generated amplicons confirmed miR106a-*RepA* duplex enrichment at four predicted miR106a MREs in *RepA* (Fig. 2C, D). To further confirm these results, we used synthetic locked nucleic acid (LNAs) to uniquely disrupt miR106a pairing at each MRE in the *RepA* region, as indicated in Fig. 2C. The H4SV cells treated with LNAs expressed X_i_-linked GFP but not the autosomal miR106a target genes (Supplementary Fig. 2F), confirming the functional role of miR106a-*RepA* pairing in transcriptional repression of X_i_-linked genes. We next quantified the enrichment of miR106a-*RepA* duplex following the PARIS2 assay, which was significantly diminished in the LNA-treated cells relative to control cells (Fig. 2E). In concert with the PARIS2 analysis, the biotinylated miR106a/RNA complex was enriched for *Xist* in miR106a mimic-transfected H4SV cells (Fig. 2F, *Left*), and reciprocally, a pull-down complex was enriched for miR106a in cells transfected with a biotinylated *Xist* probe (Fig. 2F, *Right*). Consistently, an RNA FISH analysis showed significant miR106a enrichment in the *Xist*-positive nuclear regions in mouse fibroblast cells (Supplementary Fig. 2G).

### miR106a-specific sponge stably inhibits miR106a function

XCI is a time-dependent progression of diverse epigenetic events culminating in the transcriptional silencing across X_i_. Thus, to achieve prolonged stable miR106a suppression, we engineered a synthetic oligonucleotide harboring a tandem repeat of the perfect and bulged miR106a binding, hereafter referred to as miR106a sponge (miR106sp). The in vitro binding (Supplementary Fig. 3A) and competitive elution assay (Supplementary Fig. 3B) confirmed the physical interaction between miR106a and miR106sp. Lentivirus-mediated expression of miR106sp in BMSL2 cells significantly reduced nuclear miR106a (Supplementary Fig. 3C). qRT-PCR analysis confirmed reduced *Xist* levels and derepression of X_i_-linked genes in miR106sp-expressing H4SV cells (Supplementary Fig. 3D). Notably, lentivirus-mediated expression of miR106sp in human HEK293T cells significantly reduced nuclear miR106a and *Xist* levels (Supplementary Fig. 3E), confirming the efficiency of miR106sp in both mouse and human cells.

The biological activity of miR106sp was confirmed using a luciferase reporter system, which showed a ~ 75% lower Renilla/Firefly ratio and was further augmented by the ectopic expression of miR106a (Supplementary Fig. 3F). Co-transfection of plasmids encoding a perfect miR106a target site, miR106a, and miR106sp significantly reversed miR106a-mediated suppression of luciferase activity (Supplementary Fig. 3G). Mechanistically, miR106sp-miR106a complex associates with the canonical RNA interference machinery in BMSL2 and HEK293T as determined by RNA immunoprecipitation (RIP) assay (Supplementary Fig. 3H).

### miR106a-*RepA* pairing is required for *Xist*-X_i_ association and localization

**Fig. 3:**
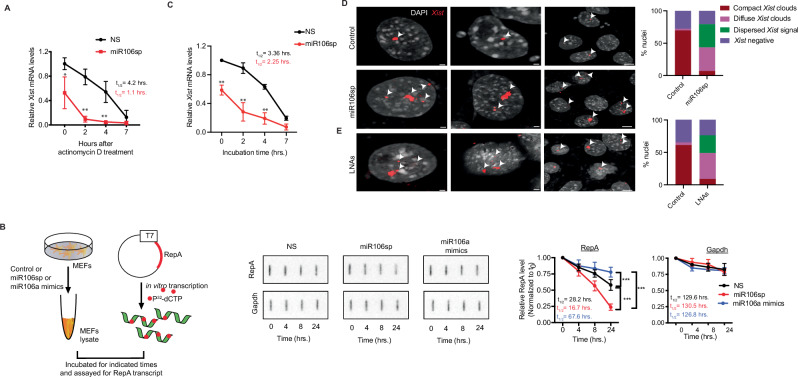
Loss of miR106a-*RepA* pairing interferes with *Xist*-X_i_ association. **A** qRT-PCR analysis monitoring *Xist* levels in H4SV cells expressing miR106sp following treatment with actinomycin D. *Gapdh* mRNA was used as a normalization control. *n* = 3. For 0 h, *p = 0.014; 2 h, **p = 0.0013; 4 h, **p = 0.0089. **B** Schematic showing the in vitro stability assay for P^32^-labeled *RepA* in whole-cell lysates expressing NS, miR106sp, or miR106a mimics (*Left*). Slot blot assay monitoring stability of in vitro synthesized uncapped *RepA* in a time-dependent manner. In vitro-synthesized *Gapdh* is used as an endogenous control. The representative images (*Middle*) and the results quantified from three experiments (*Right*) are shown. For RepA, 24 h. miR106a mimics vs. NS, ***p = 0.00049; miR106sp vs. NS, ***p = 0.00035; miR106sp vs. miR106a mimics ***p = 9.39 × 10^-6^. **C** qRT-PCR analysis of EU-labeled nascent *Xist* RNA prepared from the cells expressing control or miR106sp. *Gapdh* mRNA was used as a normalization control. *n* = 3. For 0 h, **p = 0.0091; 2 h, **p = 0.0061; 4 h, **p = 0.0052. **D, E** Confocal-airyscan sections of nuclei stained for *Xist* in control and miR106sp- (**D**) or LNAs (**E**) treated H4SV cells. The proportion of *Xist*-positive cells is quantitated (*Right*). The representative images are shown, and the results are quantified from three experiments **D, E**. Data is expressed as mea*n* ± SD (**A–C**). Unpaired, two-sided t-test with no multiple adjustments (**A–C**). Scale bars: 2 µm (D, *Left*) and 5 µm (D, *Right*).

We noted a modest but significant decrease in *Xist* levels in miR106a-depleted cells (Supplementary Fig. 2A), raising the possibility that miR106a-*RepA* could be required for *Xist* expression and/or localization to the X_i_. Interestingly, loss of miR106a did not affect RNA PolII recruitment to the Xist promoter (Supplementary Fig. 4A) but decreased the half-life (t_1/2_) of *Xist* RNA from ~4 h. to ~1.1 h. in cells depleted for miR106a (Fig.3A and Supplementary Fig. 4B). In concert with in-cell analysis, the stability of in vitro synthesized *RepA* fragment showed a gradual decrease when incubated with cell lysates expressing miR106sp compared to the control, with t_1/2_ of ~16 h and ~ 28h, respectively (Fig. 3B). A significantly higher value was obtained for the *RepA* in the presence of miR106a mimics (Fig. 3B). Given that *Xist* is a 5’ capped lncRNA, we incorporated 7-methyl guanosine (m^7^G) at the 5’-end of in vitro transcribed *RepA*. A similar trend was obtained for the 5’-capped *RepA* (Supplementary Fig. 4C). In contrast, the t_1/2_ in vitro synthesized *Gapdh* was unchanged by miR106a inhibition (Fig. 3B and Supplementary Fig. 4C). RNase treatment before incubation with cell lysate abolished the *RepA* signal (Supplementary Fig. 4C). Finally, we used an RNA pulse labeling strategy to measure the half-life of the nascent *Xist* RNA in vivo. The t_1/2_ of *Xist* was significantly reduced from ~3.0 h. to ~2.0 h. in cells treated with miR106sp (Fig. 3C).

Next, we examined the chromosome-wide accumulation of *Xist* in miR106sp-treated and control cells using RNA FISH. Intriguingly, we observed distinctly diffused staining patterns for *Xist*, occupying more nuclear space and/or punctate across the nucleus in miR106sp-treated fibroblasts (~72%; Fig. 3D) and MEFs (~66%; Supplementary Fig. 4D). In contrast, the control cells showed discrete and highly contained *Xist* clouds (~69%; Fig. 3D and Supplementary Fig. 4D). To determine if the lack of localization is due to artifacts or secondary effects created by miR106a depletion, we specifically targeted miR106a-*RepA* association via LNAs. Strikingly, RNA FISH analysis showed a distinct staining pattern characteristic of displaced *Xist* in LNA-treated cells (~67%, Fig. 3E), indicating that miR106a-*RepA* pairing is required for *Xist* association with X_i_.

### Loss of miR106a-*RepA* compromises binding of *Xist*-interacting proteins

**Fig. 4:**
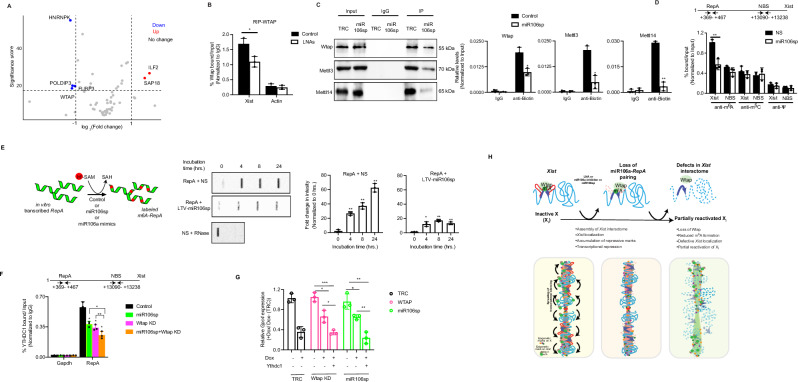
Loss of miR106a-*RepA* partnering compromises the assembly of Wtap and m^6^A formation on *Xist.* **A** Volcano plot depicting differential pull-down of 62 *Xist*-interacting proteins in control and miR106a-depleted cells. Blue indicates proteins downregulated (*n* = 4), *Red* indicates proteins upregulated (*n* = 2), and *Gray* indicates no change (*n* = 56) in miR106a-depleted cells relative to control cells. *n* = 3. **B** RIP assay monitoring binding of Wtap to *Xist* and *Actin* in control or LNAs-treated cells. *n* = 3. *p = 0.015. **C** Immunoblot of proteins retrieved by *Xist* and isogenic control probes from H4SV cells expressing NS or miR106sp by ChIRP. Input is 2% of the total protein. The representative images (*Left*) and the results quantified from three experiments (*Right*) are shown. Wtap: Control vs. miR106sp, *p = 0.032; Mettl3: Control vs. miR106sp, *p = 0.023; Mettl14: Control vs. miR106sp, **p = 0.0029. **D** qRT-PCR analysis monitoring m^6^A, m^5^C, and Ψ enrichment on *Xist* in H4SV cells expressing control or miR106sp. The predicted non-binding site (NBS) in *Xist* for m^6^A, m^5^C, and Ψ is used as a negative control. *n* = 3. For m^6^A: NS vs. miR106sp, **p = 0.0069. **E** Scheme of in vitro methylation assay with *RepA* using ^3^H-labeled SAM (*Left*). Slot blot assay monitoring methylation of in vitro synthesized *RepA* incubated with whole cell lysate prepared from cells expressing NS or miR106sp in a time-dependent manner. The representative images (*Middle*) and the results quantified from three experiments (*Right*) are shown. *RepA* pre-treated with RNase was used as a negative control. For NS: 4 h, **p = 0.0024; 8 h, **p = 0.0048; 24 h, **p = 0.0054. For miR106sp: 4 h, *p = 0.027; 8 h, **p = 0.0039; 24 h, **p = 0.01. **F** RIP assay monitoring binding of Ythdc1 to *Xist* and *Gapdh* in H4SV cells ectopically expressing NS or miR106sp or Wtap shRNA. *n* = 3. Control vs. miR106sp, *p = 0.035; Control vs. Wtap KD, *p = 0.049; Control vs. miR106sp+Wtap KD, *p = 0.033; miR106sp vs. miR106sp+Wtap KD, *p = 0.05; Wtap KD vs. miR106sp+Wtap KD, **p = 0.0037. **G** qRT-PCR analysis of pSM33 Xist-(BoxB)_3_ ES cells expressing DC1 and Wtap shRNA or miR106sp on *Xist*-mediated gene silencing. Gene expression was normalized to *Gapdh* and *Gpc4* levels prior to *Xist* induction in cells expressing empty vectors. *n* = 3. For WTAP KD, Dox^-^ vs. Dox^+^, *p = 0.012, Dox^+^ vs. Ythdc1^+^, *p = 0.014, dox^-^ vs. Ythdc1^+^, ***p = 0.00029; For miR106sp, Dox^-^ vs. Dox^+^, *p = 0.03, Dox^+^ vs. Ythdc1^+^, **p = 0.0029, dox^-^ vs. Ythdc1^+^, **p = 0.0045. **H** Schematic model of miR106a-directed regulation of X_i_ silencing. Data is expressed as mean ± SD (**B–G**). Unpaired, two-sided t-test with no multiple adjustments (**B–G**).

*Xist* localization to a X_i_ is dictated by its cis-transcriptional locus and binding of diverse proteins^10,29–31^. Significantly, the modular architecture and abundant sequence motifs in *RepA* serve as a nucleation center for multiple *Xist*-interacting proteins^32^. We, therefore, considered the possibility that loss of miR106a-*RepA* pairing induces local and higher-order structural changes that affect the binding of *Xist*-interacting proteins. To test this idea, we interrogated the effect of miR106a depletion on the protein partners of *Xist* by *Xist*-comprehensive identification of RNA binding proteins followed by mass spectrometry (ChIRP-MS). Silver staining of ChIRP samples showed that the *Xist* probe pulled down proteins, whereas RNase-treated samples were clean (Supplementary Fig. 5A). For the 81 reference proteins previously described^10^, we discovered that the binding of 4 proteins, Wtap, Hnrnpk, Poldip3, and Fubp3, was significantly decreased in miR106sp-treated cells (Fig. 4A and Supplementary Fig. 5B and Supplementary Data II). Notably, Wtap exclusively binds *RepA*^10^ and is a member of the methyltransferase complex required for m^6^A formation on *Xist* RNA^12^. RIP analysis confirmed a significant decrease in the Wtap binding to *Xist* following LNAs-mediated disruption of miR106a-*RepA* paring in H4SV cells (Fig. 4B). Consistently, the western blot analysis of proteins retrieved from miR106sp-treated cells by ChIRP assay confirmed a significant decrease in the methyltransferase complex in the *Xist* pull-down complex relative to the control (Fig. 4C and Supplementary Fig. 5C). RIP analysis also confirmed the decreased binding of Wtap, Mettl3, and Mettl14 to *Xist* RNA in miR106sp-treated cells (Supplementary Fig. 5D). Notably, no significant changes in the expression of methyltransferase complex genes were observed in cells expressing miR106sp (Supplementary Fig. 5E).

Given that the miR106a MREs were in proximity to m^6^A peaks in *RepA* (Supplementary Fig. 5F), we tested the functional implication of loss of Wtap association with *Xist* in the m^6^A formation through a series of experiments. Loss of miR106a is accompanied by decreased m^6^A formation but did not affect the other known RNA epigenetic modifications on *Xist* (Fig. 4D). In concert with in-cell analysis, the miR106a inhibition significantly reduced S-adenosyl methionine (H^3^-SAM)-dependent methylation of *RepA* transcript relative to control as monitored by in vitro methylation assay (Fig. 4E). Lack of signal following RNase treatment confirmed RNA-specific signal (Fig. 4E). We used m^6^A formation as a readout to ask whether canonical miRNA biogenesis proteins are required for miR106a function in XCI. Knocking down the *Dicer*, the endonuclease required for mature miRNA or individual *Ago* (Ago1–4) proteins, significantly reduced m^6^A enrichment on *Xist* (Supplementary Fig. 5G, H).

Finally, we assessed the effect of miR106a inhibition on the recognition of m^6^A by Ythdc1, a specific reader of m^6^A modification on *Xist*^12^. Knockdown of miR106a or Wtap diminished Ythdc1 binding to *Xist*, which was further augmented by the dual inhibition of Wtap and miR106a (Fig. 4F and Supplementary Fig. 5I). Previous studies have shown that synthetic Ythdc1 docking to *Xist* could rescue *Xist*-mediated gene silencing upon loss of m^6^A^12^. Indeed, the artificial tethering of DC1 to *Xist* partially rescued *Gpc4* silencing in inducible XIST–(BoxB)_3_ pSM33 ES cells lacking miR106a and WTAP to various extents (Fig. 4G and Supplementary 5J, K).

Together, we propose a model for miR106a function in XCI that requires miR106a-*RepA* pairing for the assembly of *Xist*-interacting proteins, such as Wtap, and loss of which displaces the *Xist* interactome, affecting *Xist-*X_i_ association, leading to the transcriptional derepression of X_i_ (Fig. 4H).

### AAV9-mediated miR106sp expression increases *Mecp2* levels in the brain and improves disease phenotypes of an RTT mouse model

**Fig. 5:**
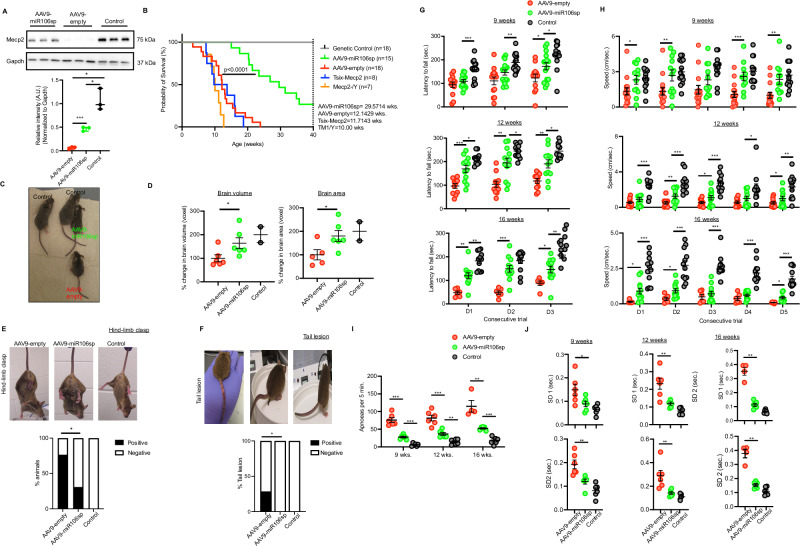
miR106sp-mediated *Mecp2* expression in the brain improves the neurobehavioral phenotype of RTT. **A** Immunoblot (*Top*) and quantitation (*Bottom*) of Mecp2 levels in the whole-brain lysates derived from the AAV9-miR106sp and AAV9-empty-injected *Tsix-Mecp2* and control mice at ~16 weeks. *n* = 3. For AAV9-empty vs. AAV9-miR106sp, ***p = 0.00068; AAV9-miR106sp vs. Control, *p = 0.015; AAV9-empty vs. Control, *p = 0.036. **B** The lifespan of AAV9-miR106sp-injected mice (*n* = 15) compared with AAV9-empty-injected (*n* = 18), control (*n* = 18), *Tsix-Mecp2* (*n* = 8) and *Mecp2*^*-/Y*^ (*n* = 7) animals. The data was analyzed using the log-rank test. **C** Representative images of AAV9-empty-injected (*n* = 1) or AAV9-miR106sp-injected (*n* = 1) or control (*n* = 2) mice at ~12 weeks of age. **D** The percent changes of in vivo MRI brain volume (*Left*) and brain area (*Right*) in AAV9-empty-injected (*n* = 6) or AAV9-miR106sp-injected (*n* = 6) or control (*n* = 2) mice at ~6 weeks of age. Control animals were used as a reference for images and were excluded from the statistical analyses. For brain volume, AAV9-empty vs. AAV9-miR106sp, *p = 0.041. For brain area, AAV9-empty vs. AAV9-miR106sp, *p = 0.035. **E, F** Representative images (*Top*) and the quantitation (*Bottom*) of AAV9-empty-injected (*n* = 17) or AAV9-miR106sp-injected (*n* = 14) or control (*n* = 18) mice showing hind-limb clasping (**E**) and tail lesions (**F**). For the hind-limb clasp, AAV9-empty vs. AAV9-miR106sp, *p = 0.033. For the tail lesion, AAV9-empty vs. AAV9-miR106sp, *p = 0.024. **G** Latency to fall (mean + /- SEM) in rotarod test at 9, 12, and 16 weeks old AAV9-empty-injected (*n* = 13) or AAV9-miR106sp-injected (*n* = 12) or control (*n* = 12) mice. Data for the three consecutive days of evaluation is shown. The data was analyzed using a Welch one-way ANOVA with Dunnett’s post hoc multiple comparisons. **H** Averaged speed in the Barnes maze test for AAV9-empty-injected (*n* = 13) or AAV9-miR106sp-injected (*n* = 12) or control (*n* = 12) mice at 9, 12, and 16 weeks. Data for the five consecutive days of evaluation is shown. **I** Apneas (>0.5 sec.) measured by whole-body plethysmograph (mea*n* + /- SEM) per 5 min as measured in AAV9-empty-injected or AAV9-miR106sp-injected or control mice at the indicated times (*n* = 6 animals per group). **J** Summary data (mea*n* + /- SD) illustrating changes in short-term (SD1) and long-term (SD2) variabilities in the respiratory cycle period for AAV9-empty-injected or AAV9-miR106sp-injected or control mice at 9, 12, and 16 weeks (*n* = 6 animals per group). p-values are included in the Source data (**G–J**). Data is expressed as mean ± SD (**A, D, G–J**). Unpaired, two-sided t-test with no multiple adjustments (**A, D, H–J**).

Previous studies showed that pharmacological or genetic manipulation of XCI regulators could induce an epigenetic state more permissive to X_i_-linked gene expression, raising the possibility of X_i_-reactivation-based therapeutic intervention for neurodevelopmental disorders, such as RTT^13,14,17,33,34^. We, therefore, interrogated whether the transcriptional state of X_i_ is sensitive to miR106a function in vivo using an adeno-associated viral vector (AAV) serotype 9 encoding a U6 promoter-driven miR106sp. 5.5 × 10^10^ viral genomes (vg) of AAV9-miR106sp or AAV9-empty particles were administered intracerebroventricularly in female *Tsix*^*ΔCpG-/+*^*:Mecp2*^*+/-*^ (referred to as *Tsix-Mecp2*^35^) mice at post-natal day 1–3. Western blot and qRT-PCR analysis of the brain tissue confirmed an increase in *Mecp2* levels in AAV9-miR106sp-injected *Tsix–Mecp2* mice, reaching ~32% of that in control animals (Fig. 5A and Supplementary Fig. 6A, B). As expected, a modest but significant decrease in *Xist* was observed in the brain tissue of AAV9-miR106sp-injected *Tsix–Mecp2* mice relative to AAV9-empty injected mice (Supplementary Fig. 6C). Furthermore, immunofluorescence analysis of brain sections from AAV9-miR106sp-injected mice revealed ~37.5% of the cells stained positively for Mecp2 (Supplementary Fig. 6D). Notably, the Mecp2 fluorescence signal intensity in the AAV9-miR106sp-injected sections was lower than for the control brain sections (Supplementary Fig. 6D, *Insets*), indicating lower levels of *Mecp2* from fewer brain cells. Likewise, the flow cytometry analysis of brain cells from ~16-week-old AAV9-miR106sp-injected *Xist*^*Δ+/-*^*:Mecp2/Mecp2-GFP*^17^ mice identified ~10–12% NeuN and GFP-positive cells but not in AAV9-empty-injected mice (Supplementary Fig. 6E).

Previous studies have shown that the Mecp2 protein rescue, either from a small number of cells expressing high-level *MECP2*^27^ or a greater number of cells expressing low-level *MECP2*^35^ or a subtype of brain cells expressing *MECP2*^36^, could reduce the disease severity. Indeed, in our study, the lifespan of AAV9-miR106sp-injected mice was significantly extended compared to AAV9-empty-injected mice, with a median survival of ~29.57 and ~12.14 weeks, respectively (Fig. 5B). Consistent with previously published studies^35^, *Tsix-Mecp2* showed a median survival of ~11.71 weeks for females (Fig. 5B). The oldest AAV9-miR106sp-injected mice reached over 50 weeks of age, and AAV9-empty-injected mice reached 23.9 weeks of age. We noted that AAV9-empty and AAV9-miR106sp-injected mice were undersized relative to control animals (Fig. 5C and Supplementary Fig. 6F).

Overall, the general appearance of AAV9-miR106sp-injected mice improved compared to that of AAV9-empty-injected mice (Supplementary Movie I). Magnetic resonance imaging revealed a significant increase in the brain volume and area of AAV9-miR106sp-injected animals relative to the AAV9-empty-injected mice, while overall anatomy did not show any differences (Fig. 5D and Supplementary Fig. 6G, H). Among the cohort of animals tested until the maximum age of AAV9-empty treated animals (~24 weeks), AAV9-miR106sp-injected animals showed a delay of hind-limb clasping (~30% vs. ~76%) and tail lesions (0% vs. ~20%) phenotypes (Fig. 5E, F and Supplementary Movie II).

AAV9-miR106sp-injected animals showed significant improvement in the general activity levels, gross locomotor activity, and exploratory behaviors relative to AAV9-empty-injected animals in an open field (Supplementary Movie III). We, therefore, longitudinally compared the neurobehavioral outcomes in the group using sensory-motor tests. The motor abilities of the cohort were assessed on the rotating rod test starting at nine weeks of age, representing the early symptomatic stage of the disease. AAV9-miR106sp-injected mice exhibited improved motor function and coordination compared to the AAV9-empty-injected mice at 12 and 16 weeks of age (Fig. 5G and Supplementary Movie IV).

The Barnes maze test evaluated exploratory behavior and efficiency to complete the task at 9, 12, and 16 weeks of age. Among the cohort of animals tested, the AAV9-empty-injected mice were least exploratory, as indicated by animal tracks (Supplementary Fig. 6I) and were least efficient in finding the target escape hole, as indicated by the reduced speed (Fig. 5H) and percent success (Supplementary Fig. 6J). Overall, AAV9-miR106sp-injected mice showed significant improvement in the exploratory behavior compared to AAV9-empty-injected mice, but did not reach the abilities of the control animals (Fig. 5H and Supplementary Fig. 6I, J).

Breathing abnormalities are another common and debilitating feature of the RTT syndrome^37^, and our whole-body plethysmography of *Tsix-Mecp2* mice identified a pronounced respiratory phenotype at ~9 weeks of age (Supplementary Fig. 6K, L). The AAV9-empty-injected mice exhibited increased apnea episodes and irregular breathing patterns (Fig. 5I, J and Supplementary Fig. 6K, L). Strikingly, a significant improvement in apneas and breathing patterns was recorded for the AAV9-miR106sp-injected mice (Fig. 5I, J and Supplementary Fig. 6K, L).

### miR106sp-mediated *MECP2* expression in RTT neurons improves the morphological and calcium transient deficits

**Fig. 6:**
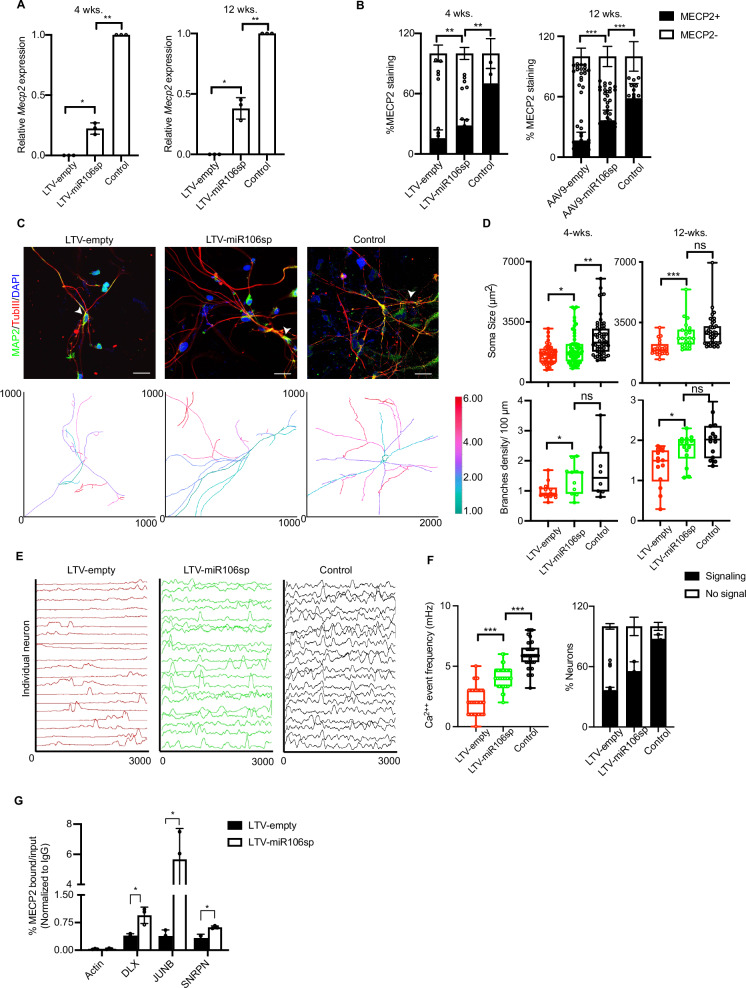
miR106sp-mediated *MECP2* expression in RTT neurons improves the morphological and calcium transient deficits. **A** qRT-PCR analysis monitoring the expression of X_i_-linked *MECP2* in 4- and 12-week-old RTT neurons infected with LTV-empty or LTV-miR106sp. Neurons derived from a clone expressing wild-type *MECP2* from the X_a_ were used as a positive isogenic control (Control), which was set to 1. *n* = 3. For 4 weeks, LTV-empty vs. LTV-miR106sp, *p = 0.014; LTV-miR106sp vs. Control, **p = 0.0012. For 12 weeks, LTV-empty vs. LTV-miR106sp, *p = 0.018; LTV-miR106sp vs. Control, **p = 0.0068. **B** Quantification of 4- and 12-week-old LTV-empty or LTV-miR106sp-infected RTT-neurons and control neurons with nuclear MECP2 immunostaining. *n* = 200 cells per group in three independent experiments. For 4 weeks, LTV-empty vs. LTV-miR106sp, **p = 0.0075; LTV-miR106sp vs. Control, **p = 0.0046. For 12 weeks, LTV-empty vs. LTV-miR106sp, ***p = 4.77 × 10^-05^; LTV-miR106sp vs. Control, ***p = 0.00011. **C** Representative images of MAP2 (*Green*) and TubIII (*Red*) staining in ~4-week-old LTV-empty or LTV-miR106sp-infected RTT- and control neurons. DAPI stains the nucleus Blue (*Top*). Representative reconstructed neuronal images for the quantitative assessment of all orders of branches in each group by Sholl analysis are shown (*Bottom*). **D** Quantitative analysis of the soma cross-sectional area (*Top*) and the number of neuronal branch points (*Bottom*) in 4- and 12-week-old MAP2 + RTT neurons expressing empty or miR106sp and control neurons. *n* = 200 cells per group in three independent experiments. The boxed areas span the first to the third quartile, with the central line representing the median expression changes for each group. Soma size: For 4 weeks, LTV-empty vs. LTV-miR106sp, *p = 0.024; LTV-miR106sp vs. Control, **p = 0.001. For 12 weeks, LTV-empty vs. LTV-miR106sp, ***p = 0.00039; LTV-miR106sp vs. Control, ^ns^*p* = 0.425. Branches density: For 4 weeks, LTV-empty vs. LTV-miR106sp, *p = 0.015; LTV-miR106sp vs. Control, ^ns^*p* = 0.491. For 12 weeks, LTV-empty vs. LTV-miR106sp, *p = 0.015; LTV-miR106sp vs. Control, ^ns^*p* = 0.142. **E, F** Representative fluorescent intracellular calcium transient in RTT neurons expressing empty or miR106sp or control neurons over 5 min (*n* = 20; **E**). The quantitation of calcium spikes for each group is shown (**F**, *Left*). The boxed areas span the first to the third quartile, with the central line representing the median expression changes for each group. For calcium event frequency, LTV-empty vs. LTV-miR106sp, ***p = 7.43 × 10^-13^; LTV-miR106sp vs. Control, ***p = 1.09 × 10^-11^. The percentage of GCaMP6-positive neurons in each group is shown (F, *Right*). (*n* = 60 per group) **G** ChIP analysis monitoring binding of MECP2 to the Actin, DLX (LTV-empty vs. LTV-miR106sp, *p = 0.049), JUNB (LTV-empty vs. LTV-miR106sp, *p = 0.040), and SNRPN (LTV-empty vs. LTV-miR106sp, *p = 0.026) promoter in RTT neurons expressing control or miR106sp. Data is expressed as mea*n* ± SD (**A, B, F, G**). Unpaired, two-sided t-test with no multiple adjustments. Scale bars: 40 µm (**D**).

We next asked whether miR106sp-mediated wild-type *MECP2* could normalize the morphological defects in human postmitotic neurons, the cell type most relevant to RTT. To obtain RTT neurons, we used a previously described clonal induced pluripotent stem cell (iPSC) line, T158M-iPSC, which carries mutant *MECP2* on the X_a_chromosome and wild-type *MECP2* on the X_i_ chromosome^38^. As a positive isogenic control, we used a non-RTT iPSC clone, which carries wild-type *MECP2* on the X_a_. Neuronal differentiation was initiated to produce neuronal precursor cells, which were then differentiated into neurons^17^. miR106a inhibition in RTT neurons expressed X_i_-linked wild-type *MECP2* at four weeks, which was further augmented by 12 weeks (Fig. 6A). Consistently, RTT neurons expressing miR106sp stained positively for MECP2 relative to empty vector expressing neurons (Fig. 6B and Supplementary Fig. 7A).

miR106a inhibition significantly improved the soma size and branch density in MAP2^+^ RTT neurons relative to neurons transduced with empty vector (Fig. 6C, D and Supplementary Fig. 7B). Given that disturbances in calcium homeostasis are associated with MECP2 loss in the murine model^39^, we analyzed calcium-dependent neuronal networks in GcaMP6-loaded RTT neurons in the context of miR106a function. Strikingly, the miR106sp-mediated *MECP2* expression significantly increased the calcium event frequency in RTT neurons (Fig. 6E, F).

To substantiate the biological function of miR106sp-mediated MECP2, we analyzed genomic loci previously identified as brain-specific MECP2 targets^40^, including DLX, JUNB, and SNRPN. As expected, RTT neurons expressing miR106sp showed an overall increase in MECP2 enrichment at the indicated gene loci relative to empty vector-expressing neurons as measured by directed-ChIP assay (Fig. 6G).

## Discussion

In mammals, a vast majority of transcripts are non-coding RNAs, including lncRNA, miRNA, Piwi-interacting RNAs, transcription initiation RNAs, and small nucleolar RNAs. Generally, these RNA species interact with DNA, RNA, and proteins to regulate gene expression by remodeling chromatin, altering transcriptional activity, or changing protein stability^41,42^. More recently, lncRNA and miRNA interactions were identified in human cancers and cardiovascular diseases, unraveling the clinical significance of the lncRNA-miRNA partnering^43^. As a result, new non-canonical functions are attributed to miRNAs in lncRNA stability^44^, using lncRNA as a decoy^44,45^, competitive mRNA binding^46^, and biogenesis from lncRNA^47^. Despite miRNA abundance on the X chromosome^48,49^, no function for miRNAs in XCI has previously been demonstrated.

Here, we performed a large-scale genome-wide CRISPR/Cas9 screen to identify the miRNAs required for mammalian XCI. The validation of miRNA candidates confirmed that their knockdown is sufficient to disrupt the transcriptional silencing of the X-linked genes. Using PARIS2, we captured and mapped miR106a binding sites across the *RepA* region of *Xist*. We uniquely used LNAs to target the miR106a-*RepA* interaction, which de-repressed X_i_-linked genes but did not affect non-X targets of miR106a. Our results bode well with previous studies where LNAs targeted at the Repeat C domain displaced *Xist* RNA and affected PRC2 complex recruitment^50^. Through functional analysis, we demonstrated that loss of miR106a affects the assembly and composition of *Xist*-interacting proteins, such as Wtap, which is accompanied by defects in *Xist* localization to X_i_ and gene silencing. Consistent with previously published studies^12^, we find that synthetic DC1 docking to *Xist* overrides the miR106sp-mediated Wtap/m^6^A deficit and partially rescues *Xist*-mediated transcriptional silencing of X_i_-linked genes.

It is remarkable that targeting a small region harboring the miR106a:*RepA* interaction of around 10–20 bp within a 15 kb RNA can have long-ranging effects on the transcriptional status of X_i_. These dramatic effects could be driven by miR106a-mediated local and higher-order structural changes required for *Xist* interacting proteins. Importantly, the modular architecture of repeat elements in *RepA* determines the specificity of RNA-binding protein^6,10^ and, thus, shapes the *Xist* interactome. For example, the secondary structure of A repeat was critical for the binding and clustering of Spen into a higher-order structure^51^. Intriguingly, the pharmacological alteration of the *RepA* structure causes X_i_-specific loss of polycomb complex and H3K27me^3^ enrichment and failure of XCI^8^. Unlike other regions of *Xist*, the *RepA* region is uniquely structured with preferential inter-domain folding rather than intra-repeat duplexes^32^.

Given the significance of expressing X_i_-linked genes by epigenetic reprogramming, the discovery of miR106a-mediated regulation of XCI provides a therapeutic opportunity for X-linked neurodevelopmental disorders, including but not limited to RTT (reviewed^25^). Importantly, this approach expresses genes from an endogenous locus alongside the cis-regulatory elements and maintains the local chromosomal context, which could be critical for clinically beneficial gene dosage in RTT girls. Another key consideration is the efficacy and tolerability of this approach in humans, which needs to be systematically evaluated. Critically, the miR106sp-mediated XCI defect is expected to be equivalent in healthy and mutant cells, which could cause healthy cells to express mutant *MECP2*. However, more than 60% of RTT-causing mutations are nonsense or frameshift that would give rise to a premature stop codon, i.e., these *MECP2* transcripts are degraded by nonsense-mediated mRNA decay^52^. In addition, 35% of the missense mutations map to the DNA-binding domain of *MECP2* and reduce binding to methylated DNA by more than 100-fold^53^. Finally, the wild-type form is dominant in cells expressing wild-type and mutant *MECP2* in vivo^36^. Thus, the possibility of a truncated form of protein that retains partial function is highly unlikely.

We find that long-term, stable miR106sp treatment expresses *MECP2* at ~20–30% of that expressed in healthy neurons or the brain of a mouse (set to 100%) and increases the survival of the RTT mice. However, additional effects of targeting miR106a on gene expression and dosage compensation cannot be ruled out. We note that several FDA-approved drugs, such as *Tazemetostat*^54^, *Imatinib*^55^, *Metformin*^56^, and *Aspirin*^57^, induce multifactorial changes in gene expression and yet are well tolerated in humans^58,59^. Nonetheless, it is critical to survey the gene expression changes for X-linked genes following miR106sp treatment. Overall, the miR106sp-treatment of RTT neurons did not significantly change the total X-linked and autosomal gene expression relative to control cells (Supplementary Fig. 7C). The gene expression analysis by binning fold change values in increments of 0.2 showed an average fold change of 1.08-fold for X-linked genes and 1.06-fold for autosomal genes for RTT neurons expressing miR106sp (Supplementary Fig. 7D). Interestingly, only 7.86% of X-linked genes were up-regulated greater than 1.5-fold, which is significantly lower than the X-linked gene alterations reported for *Xist* deletion^13,60^. Consistent with our results, multiple studies have previously reported that the general X_i_-reactivation does not cause robust gene expression changes^13,33,60^ or in vivo consequences in the context of phenotype and morbidities (reviewed^25^). A likely explanation for the apparent safety of the general X_i_ reactivation could be the inherent tolerance to an unexpectedly large variation in the gene expression profiles (~83%) among individuals^61^. Furthermore, over 25% of human X-linked genes are expressed biallelically due to incomplete XCI^62–64^. Notably, the allele-specific expression estimates revealed variable expression of escape genes, averaging around 10–33% of X_a_ expression in tissues and individuals^64^, highlighting the in-between-female diversity introduced by incomplete XCI and tolerability of the gene expression changes.

### Limitations of the study

While we identify a previously unknown regulator of *Xist*-X_i_ pairing required for the inactivation of the X chromosome, our understanding of the mechanism by which miR106a exerts its effects on X_i_ transcription remains limited. We speculate that miR106a could drive the conformational changes in *Xist* that are critical for its localization and interaction with other proteins, such as WTAP. However, further investigations are warranted to determine the role of miR106a in either facilitating the folding of *RepA* as an isolated unit or preventing the interaction with other domains. Another limitation is that we have only measured the effects of miR106a depletion on the expression of selected genes from X_i_. Prior to further advancing AAV9-miR106sp to the clinic, safety studies will be required to further evaluate the therapeutic profile and dosing window.

## Methods

Mouse husbandry and experiments were carried out under the guidance of the University of Virginia and the University of California, Davis Animal Care and Use Committee (IACUC). Work involving mice adhered to the guidelines of the University of Virginia, IACUC protocol number 4112, and the University of California, Davis, IACUC protocol number 24090. Animal care was provided in accordance with the procedures outlined in the Guide for the Care and Use of Laboratory Animals.

### Cell lines and cell culture

H4SV cells, BMSL2 (HOBMSL2) cells, and HEK293T cells were cultured as previously described^13^. NIH3T3-rtTA-IRES-BSD, provided by Takashi Sado (Kyushu University), were cultured as previously described^65^. pSM33 ES cells expressing *Xist*-(BoxB)_3_ were provided by Mitchell Guttman (California Institute of Technology) and were maintained as previously described^12^. Cells cultured simultaneously were pooled and then seeded after counting in a 6-well or 10-cm dish. In random order, cells were then treated with control or different biologics, including shRNA, sgRNA, synthetic inhibitors, and vectors. Cells were routinely tested for mycoplasma using the PlasmoTest kit from (Invivogen).

### Mice breeding and genotyping

All mice were bred and maintained in the University of Virginia and the University of California, Davis animal facility in accordance with the guidelines of IACUC. All mice were exposed to a 12:12 h. light-dark cycles with food and water administration *ad libitum*. Animals were randomly assigned to experimental groups which were non-blinded, and no specific method was used to calculate sample sizes. Mouse strains used in the study were obtained as follows: *Xist/ΔXist (B6;129-Xist<tm5Sado* > , provided by Antonio Bedalov, (Fred Hutchinson Cancer Center, Seattle), *B6.129P2(C)-Mecp2tm1.1Bird/J* (#003890, The Jackson Laboratory), *Mecp2*^*t3.1Bird*^*/J*) (#014610, The Jackson Laboratory), and *Tsix*^*ΔCpG/+*^, provided by Jeannie Lee, (Massachusetts General Hospital, Boston). *Tsix*^*ΔCpG/+*^*Mecp2*^*+/-*^ female mice were generated by crossing male *Tsix*^*ΔCpG/Y*^ mice with female *Mecp2*^*+/-*^, and *Xist*^*Δ+/-*^*:Mecp2/Mecp2-GFP* mice were generated by crossing male *Xist:Mecp2-GFP/Y* with female *XistΔ:Mecp2/Xist:Mecp2*. As described previously^17^, the animal genotype was confirmed using gene-specific primers listed in Supplementary Data III.

### Pooled genome-wide CRISPR screen

The GeCKO v2 libraries (3^rd^ generation, Addgene) in a 2-vector format were obtained through the University of Massachusetts Medical School RNAi Core Facility. The two-pooled mouse GeCKO v2.0 lentivirus library encodes 130,209 sgRNA targeting 20,611 protein-coding genes (with six sgRNA per gene) and microRNAs (4 sgRNAs per miRNA). Cas9-expressing BMSL2 cells (1.1 × 10^6^) were transduced at a multiplicity of infection of 0.2 with the two lentiviral pools, generated as previously described^13^. Following infection, cells were selected for puromycin resistance for 7 days to enrich for infected cells. Cells were HAT selected, and the candidate sgRNA was identified by Mi-Seq. To validate the candidates, 3 × 10^5^ H4SV or BMSL2 cells were transduced with single sgRNAs (Supplementary Data III) and puromycin selected for four days. For HAT selection, 3 × 10^5^ cells were plated in six-well plates and selected in a medium containing 1× HAT (Gibco) for one wk., as described previously^17^.

### RNA interference

For stable shRNA and sgRNA knockdowns, cells were seeded in a six-well plate to 60–80% confluency and subsequently transduced with 200–500μl lentiviral particles expressing shRNAs (obtained from Open Biosystems/Thermo Scientific through the UMMS RNAi Core Facility, listed in Supplementary Data III) or sgRNA in a total volume of 1 ml of appropriate media supplemented with ~6–10 µg/ml polybrene. Media was replaced after overnight incubation, and cells were subjected to puromycin selection (1 µg/ml) for at least three days, as described previously^17^. For miRNA inhibitors and mimics, cells were transfected with DharmaFECT (Horizon Discovery) transfection reagent.

### CRISPR/Cas9 targeting

The sgRNA sequences were cloned in pLentiCrispr v2 plasmid (Cas9, Addgene) and packaged into the virus as above. Cells were infected with the packaged virus, as described previously^13^, and selected with puromycin for four days.

### Quantitative RT-PCR (qRT-PCR)

Total RNA was isolated and reverse transcribed using Superscript II Reverse Transcriptase (New England BioLabs Inc.). Quantitative real-time PCR was performed as described previously^17^ using primers listed in Supplementary Data III. Gene expression was normalized to *Gapdh*. Controls lacking reverse transcriptase were carried out in parallel to rule out the possibility of DNA contamination. Each sample was analyzed at least three independent times, and the results from at least three different biological triplicates were presented.

### Vector construction

The miRNA sponge sequence for miR106a-5p was designed using miRNAsong^66^. The sequence included a tandem repeat of the perfect and bulge miR106a binding site, separated by a spacer sequence, AGTTA. The sponge sequence was cloned into pLKO.1 (Addgene) and psiCHECK (Promega) vectors. The miR106a was sub-cloned into the vector pLKO.1puro (Addgene) and the miR106a perfect binding sites were cloned in psiCHECK (Promega). All the constructs were verified by full-length sequencing.

The miR106sp expression cassette was subcloned into scAAV9.Stuffer plasmid. The scAAV9.miR106sp vector (AAV9-miR106sp) was produced at SAB Tech Inc. (Philadelphia, PA) by transient transfection procedures using a double-stranded AAV2-inverted terminal repeat (ITR)-based miR106sp vector, with a plasmid encoding the Rep2Cap9 sequence, as previously described^67–69^, along with an adenoviral helper plasmid pHelper (Stratagene, Santa Clara, CA) in HEK293T cells. The purity and titer of the vector were assessed by silver staining analysis.

### Intracerebroventricular injections

Female mice received a single intracerebroventricular (ICV) injection of AAV9-miR106sp or AAV9-Empty vector at post-natal days 1–3 following hypothermic sedation. Based on long-lasting AAV dosing experience^67–69^, we used 5.5xe10 vg per animal. This route of delivery and dose allows widespread targeting throughout the nervous system in disease-relevant cell types^67–69^. Animals were monitored continuously until fully recovered from sedation and daily after that.

### RNA immunoprecipitation (RIP) assay

RNA immunoprecipitation was performed as described previously^70^. Antibodies directed against biotin (Abcam), Ago2 (EMD Millipore), YTHDC1 (Cell Signaling), and IgG (EMD Millipore) were used to precipitate RNA. The captured RNA was analyzed by qRT-PCR. For *Xist* RIP, cells were transfected with Magna ChIRP Xist lncRNA probes (Millipore Sigma), and RNA was precipitated using a biotin antibody (Abcam). The captured miRNA was analyzed by qRT-PCR as described below.

### miRNA purification

Total miRNA was purified using the *mirVana* miRNA isolation kit (Thermo Fisher) according to the manufacturer’s instructions.

### In vitro *RepA* synthesis

The RepA fragment was amplified using female genomic DNA prepared from H4SV cells. The *RepA* fragment was prepared using a MEGAscript T7 kit (Invitrogen) in the presence of dCTP, [α^32^-P] (PerkinElmer Life Sciences). The capped *RepA* fragment was prepared using mMESSAGE mMachine T7 Transcription Kit (Invitrogen) in the presence of dCTP, [α^32^-P] (PerkinElmer Life Sciences).

### *RepA* binding assay

Total RNA samples were fragmented with RNA fragmentation buffer (100 mM Tris, 2 mM MgCl_2_) at 95 °C and chilled immediately. Fragmented RNA was incubated with antibodies directed against m^6^A (Abcam), and IgG (Millipore Sigma) was used to precipitate RNA and quantitated by qRT-PCR using specific primers (Supplementary Data III). Approximately 10% of the starting material was used as Input.

### Locked nucleic acid (LNA) nucleofection

Cy3-labeled *Xist* and control (scrambled) LNAs^13^ were added to 10^4^ H4SV cells at a final concentration of 1 µM in OptiMem using Lipofectamine (Invitrogen) every 6–8 h for 24 h.

### Competitive elution assay

The cell lysates were prepared from mouse embryonic fibroblast cells using standard methods described previously^13^. Cells were transfected with miRNA106a mimics (Dharmacon) and then immobilized on streptavidin Dynabeads (Thermo Fisher). Immobilized miR106a mimics were incubated with either in vitro synthesized *RepA* or *miR106sp*, followed by elution with capture oligonucleotides (Supplementary Data III). The pooled washes and Input were resolved on 6% native gel and imaged by a Typhoon Laser Scanner (Cytiva Life Sciences).

### Subcellular fractionation

Nuclear and cytoplasmic extracts were prepared as described previously^65^. Briefly, cells were lysed in cold NP-40 lysis buffer (10 mM Tris-Cl pH 7.5, 10 mM NaCl, 3 mM MgCl_2_, 0.5% NP-40). The lysates were centrifuged to obtain a nuclear pellet. To prepare cytoplasmic RNA, the supernatant was treated with 200 μg/mL of proteinase K in 0.1 M Tris-Cl pH 7.5, 0.22 M NaCl, 1% SDS, 12.5 mM EDTA for 60 min. at 37 °C and subsequent extraction with phenol/chloroform (Invitrogen) and ethanol (Fisher Scientific) precipitation. The nuclear and cytoplasmic RNA was prepared using TRIzol (Invitrogen).

### S-adenosyl methionine (SAM) methyltransferase assay

The whole cell lysates from cells expressing NS or miR106sp were prepared as described above. 5 µg of cell lysate was incubated with 125 µg of in vitro synthesized *RepA*, 3 µCi of ^3^H S-adenosyl methionine (PerkinElmer Life Sciences), and 0.25 µM METTL3/14 protein (Abcam) were incubated at 30 °C. The methylated *RepA* was measured by slot blot assay (AlphaMetrix Biotech) and then imaged by Typhoon Laser Scanner (Cytiva Life Sciences).

### m^6^A-, m^5^C, and ψ-qRT-PCR assay

The m^6^A-, m^5^C-, and **ψ** qRT-PCR assays were performed as previously described^71^. Poly-adenylated RNA was extracted using an mRNA Purification Kit (Thermo Fisher). About 1 µg mRNA was incubated with either an anti-m^6^A antibody (Abcam), anti-m^5^C (Cell Signaling) **ψ**, anti (Diagenode), or IgG (Millipore Sigma). The immunoprecipitated RNA was analyzed by qRT-PCR. Approximately 50 ng of mRNA was used as Input.

### miRNA-qRT-PCR a×ssay

The expression level of miRNAs was confirmed by qRT-PCR using miRNA-specific primers (Supplementary Data III). Total RNA was isolated and reverse transcribed using an All-in-one cDNA synthesis kit (Applied Biological Materials) according to the manufacturer’s protocol. U6 (Applied Biological Materials) was used as an internal control.

### Colony formation assay

Cells were plated in 6-well plates after the indicated treatment and cultured until visible colonies were observed. Colonies were fixed (100% methanol (Fisher Chemical), 37 °C) and stained with 0.1% crystal violet (Sirchie) dissolved in 20% methanol (Fisher Chemical) /80% PBS (Gibco). Cells were imaged using a ChemiDoc Imaging System (BioRad), and colonies were counted as described previously^17^.

### HAT assay

The HAT selection assay was performed, and colonies were stained with crystal violet, as described previously^17^. Briefly, HAT selection medium was added to the cells, and cells were cultured for seven days with medium replacement every three days. Following selection, surviving colonies were fixed and stained with crystal violet for visualization.

### Directed-ChIP assays

ChIP assays were performed as described previously^13^ using extracts prepared ~7 d post-retroviral transduction and puromycin selection. The DNA-protein complexes were precipitated using antibodies against POL2 (Abcam), Mecp2 (Cell Signaling), and IgG. Primer sequences used for amplifying immunoprecipitated DNA are listed in Supplementary Data III.

### RNA sensor assay

RNA sensors were designed as previously described^72^. Briefly, RNA sensors encoding miR106a in the SL P3 domain and miRNA106a MRE harboring the *RepA* sequence in the SL P4 domain were engineered. The sequences are provided in the Supplementary Data III. The buffer control or RNA sensor was incubated with DFHBI (Tocris) at 37°C for 1 hour. The fluorescence signal was recorded with the following measurement parameters: excitation wavelength = 460 nm, emission wavelength = 501 nm, slit widths = 10 nm.

### Immunoblotting

Cell extracts were prepared by lysing the cell pellet in RIPA buffer supplemented with 1 mM sodium ortho-vanadate and 10 mM PMSF. Brain samples were harvested with transcardial perfusion using ice-cold PBS. The brain tissue was homogenized in 10% SDS buffer. Immunoblots were probed using antibodies against histone H3 (Abcam), Gapdh (Thermo Fisher), METTL3 (Abcam), METTL14 (Abcam), and WTAP (Abcam).

### Chromatin isolation by RNA purification (ChIRP)

The methyltransferase protein complexes bound to *Xist* were analyzed by ChIRP in H4SV cells expressing control or miR106sp. Briefly, biotinylated *Xist*-specific probes (Millipore Sigma) were used to hybridize to *Xist* and isolate chromatin-protein complexes as described previously^10^.

### RNA FISH

RNA FISH experiments were performed using a cDNA template or ViewRNA ISH probe (Thermo Fisher) as described previously^13^. Cells were visualized on a Zeiss AxioObserver Live-Cell microscope, and images were adjusted for contrast and brightness using AxioVision Software. For quantification, ~200–500 cells from at least 10 different fields were counted and scored; only cells with a detectable RNA FISH signal were included in the analysis. All RNA FISH experiments were performed at least three times, and almost 200 cells (~20 cells per 25 field) were used for quantification.

### Airyscan microscopy

Airyscan imaging was performed with a confocal laser scanning microscope, Zeiss LSM 980 (Carl Zeiss AG, Oberkochen, Germany), equipped with an Airyscan detection unit. To maximize the resolution enhancement, an alpha Plan-Apochromat 63X oil immersion objective (Zeiss) was used. The gain for detectors and pixel dwell times was adjusted for each dataset, maintaining the lowest values to avoid saturation and bleaching effects. The Zen Black 2.1 (Version 13.0.0.0) software was used to process the acquired datasets. The software processes each of the 32 Airy detector channels separately by performing filtering, deconvolution, and pixel reassignment to obtain ~20 z-stack images. Images were processed for enhanced spatial resolution and improved SNR by performing filtering, Wiener filter deconvolution, and pixel reassignment. The Airyscan Processing Baseline Shift and the 2D or 3D reconstruction algorithm were used at the default settings. The experiments were performed at least three times, and almost 60 cells (~3 cells per 20 fields) were used for quantification.

### DNA FISH

DNA FISH experiments were performed using a mouse Chromosome X probe (Cell Line Genetics Inc.) as described previously^13^. Cells were visualized on a Zeiss AxioObserver Live-Cell microscope, and images were adjusted for contrast and brightness using AxioVision Software. For quantification, ~500 cells from at least 10 different fields were counted and scored; only cells with a detectable DNA FISH signal were included in the analysis. All DNA FISH experiments were performed at least three times, and almost 500 cells (~20 cells per 25 fields) were used for quantification.

### *Xist* stability assay

The assay was performed in cells expressing control or miR106sp or transfected with miR106a mimics (Dharmacon) or miR106a inhibitor (Millipore Sigma) as described previously^13^. After treatment with DNase (Ambion), strand-specific *Xist* RNA levels and, as a control, *Gapdh*, were quantified by qRT-PCR (Supplementary Data III).

### Electrophoretic mobility shift assay (EMSA)

For the binding between miR106sp and miR106a, the α^32^P-dCTP-labeled miR106sp was used as a probe for EMSA with unlabeled single-stranded miR106a (Dharmacon). The specificity of miRNA106a binding in the gel shift assay was confirmed by co-incubation with miRNA106a negative control (Dharmacon).

For the binding between single-stranded miR106a (Dharmacon) and RepA, radiolabeled RepA probes (20,000 CPM [^32^P]) were incubated for 30 min. at 37˚C with the corresponding miR106a as described previously^73^. Binding reactions were tested in a 6% polyacrylamide gel.

### Binding motif analysis

The mature miRNA sequences were downloaded from miRBase^74^, and the motifs were identified using HOMER^75^. To interrogate miRNA pairing with m^6^A peaks in *Xist*, the data were extracted from previously published studies^12,76,77^ and paired with the miRNA sequences using Miranda software^78^.

### DC1–λN–XIST–(BoxB)3 RNA tether function assay

The artificial DC1 tethering assay for *Xist* was performed as described previously^12^. Briefly, mouse pSM33 cells expressing Xist–(BoxB)_3_ RNA under doxycycline control were infected with lentivirus expressing control, miR106sp, Rbm15 shRNA, or Mettl3 shRNA. The cells were selected with 0.25 μg/ml puromycin for two days and transfected with 2.50 μg of pCAG-GW-hYTHDC1-λN-3×Flag-BSD plasmid using Lipofectamine transfection reagent. The *Xist* expression was induced with 2 μg/ml doxycycline, and *Gpc4* expression was measured by qRT-PCR using specific primers (Supplementary Data III).

### Mouse brain immunofluorescence

Brain samples were harvested with transcardial perfusion using ice-cold 10% Formalin (Epredia). The whole brain was embedded and sliced in an OCT compound (Fisher HealthCare). A 1:1000 dilution of the primary antibody was diluted in IF staining buffer (Cell Signaling). High-resolution confocal-like microscopy was performed using a Zeiss Axio Imager M2 Microscope equipped with an Apotome 2.0 and a 63x oil objective lens with a 1.4 numerical aperture. Images were acquired using a digital MRm camera. Exposure times and illumination intensity were kept consistent for analysis within the experiment. Single optical sections were captured and tiled across the full coronal plane to map the entire coronal section of the brain. Images were captured using a single z-stack plane, approximately 3–5 µm deep into the tissue, kept at the same depth throughout the entire map. Images were stitched together to create whole montage images that were subsequently analyzed.

### Open field test

The open-field test was used to test the exploratory behavior and general activity of mice, as previously described^79^. The mice were placed in the arena, allowed to explore it for ~5 min, and video recorded.

### Rotarod test and data analysis

The rotarod test on animals was performed as described previously^35^. Briefly, the mice were tested on a commercial rotarod apparatus (MAZE Engineers) by placing a mouse on rotating drums (3.9 cm diameter). The rod was accelerated gradually from 4–40 rpm over a 5-min trial. The animals were tested in three trials with 15-min intervals for three consecutive days.

### Barnes maze and data analysis

The Barnes maze test was performed as described^80^ at 9, 12, and 16 weeks of age. The mice were habituated to the maze for 2 days before the testing. Habituation was performed by placing mice in the middle of the maze and leaving them to explore for 5 min. Testing started on day one of the test week and continued for five consecutive days. During testing, mice were placed in the middle of the maze and given 300 seconds to find the escape hole. Each trial ended when the mouse entered the escape hole and stayed in the vicinity of the escape hole (defined by a circle of 5 cm diameter around the escape hole) for five consecutive seconds, or the 300-second maximum had elapsed. All trails were recorded and analyzed using the tracking software Ethovision 13 (Noldus, Leesburg, VA, US).

### Plethysmography and data analysis

Plethysmography was performed as previously published^81^ at 9, 12, and 16 weeks of age. Animals were placed individually into 1000 cm^3^ Plexiglas chambers (Buxco) and allowed 15 min to acclimate. The chamber was continuously flushed with dry room-temperature air (24  ±  0.5 °C) delivered by three computer-driven mass-flow regulators connected to pure O_2_, N_2_, and CO_2_ (total flow: 1 L.min^−1^). The respiratory flow signal was recorded with a differential pressure sensor, amplified (500x and band-pass filtered (1–100 Hz), and collected and analyzed using Spike 2.9.13 (Cambridge Electronic Devices).

On the day of experiments, breathing parameters were recorded for 2–4 h or until sufficient periods of quiet breathing were collected for analysis. We calculated the following breathing parameters from periods free of grooming, sniffing, and movement: respiratory cycle period (T_total_, msec.), expiratory cycle period (T_e_, msec.), and the incidence of apnea (T_e_ > 0.5 sec.).

To assess respiratory variability, breath-to-breath intervals for 100 consecutive breaths were plotted on an X-Y plot, with the X-axis reflecting the N interval and the Y-axis reflecting the *n* + 1 interval, as described previously^82,83^. Short-term variability (SD1) and long-term variability (SD2) were calculated as an index of breathing variability using the formulas described previously^84^.

### MRI and data analysis

Brain MRIs were performed on anesthetized mice (6 weeks of age) using a 1 Tesla M7 (Aspect Imaging) small-animal scanner. Mice were placed in an induction chamber and anesthetized with isoflurane/O_2_ at ~ 2.0-% vol/vol for ~5 min. The anesthetized animals were placed on the bed with their head in the 23 mm diameter mouse head coil. Mice were monitored for body temperature and respiration, with the isoflurane maintenance dose being adjusted to obtain respiration of 60–100 breaths per minute. A T2-weighted scan was obtained using a 3D Fast Spin Echo (3D-FSE) scan. 3D-FSE scan parameters resulted in an approximately 23-min scan: TR 2500 ms, effective TE 67.81 ms, Echo train length 14, Echo spacing 8.476 msec., pixel bandwidth 264.55. The matrix size of the image was 126x126x30 with a spacing of 0.16 mm x 0.16 mm x 0.3 mm. Mouse brain DICOM images were imported into the open-access 3D Slicer software for tissue segmentation and analytics.

### Non-invasive cardiac electrophysiology (ECG) and data analysis

This test captures noninvasive electrocardiogram data from conscious mice. After a 30-min acclimation period, the mouse (9, 12, and 16 weeks of age) was placed on an electrode-fitted platform and further acclimatized for 10 min before data collection. Once the animal was acclimated to the platform, it was placed in an acrylic housing chamber that contains three gel-coated footpad wells. The cardiac signals were recorded and analyzed using ECGenie Instrument (Mouse Specifics Inc., Framingham, MA). This method takes ECG recordings passively through the feet of the mice and requires them to stand 2–3 ft., touching the recording pad to obtain adequate and continuous signals. Signals were analyzed using e-Mouse (Mouse Specifics, Inc., Boston, MA), a software that analyzes heart rate, heart variability, and P, Q, R, S, and T waves for each beat.

### Neural precursor cell culture

NPC cell culture was performed as previously published^17^. RTT NPCs were provided by Maria C.N. Marchetto and Fred H. Gage, The Salk Institute for Biological Studies, La Jolla, and were infected with lentivirus expressing miR106sp as described previously^17^.

### Calcium transient analysis

The calcium imaging in RTT neurons was performed as previously published^85^. Briefly, neurons were infected with a packaged pGP-AAV-syn-jGCaMP7s-WPRE plasmid (Addgene #104487-AAV9) one week before testing. The neurons were kept in room temperature artificial cerebrospinal fluid (ACSF) containing: 3 mM KCl, 140 mM NaCl, 10 mM HEPES, 10 mM Glucose, 2 mM MgCl_2_, 2 mM CaCl_2_. The solution was bubbled with 100% O_2,_ and the pH was set by adding varied amounts of KOH. Fluorescence signals were measured with a spinning disk confocal microscope outfitted with an sCMOS Camera (ORCA-Flash4.0, Hamamatsu, Bridgewater, NJ, USA).

### Statistical analysis

To achieve statistical significance, all experiments were collected from experiments performed in technical triplicate; each experiment was repeated at least three times, and statistically significant results were obtained in independent biological replicates. Differences between groups were assayed using an unpaired, two-sided *t*-test. Comparisons between multiple treatment groups were made by one-way or two-way ANOVA with indicated multiple comparisons *post hoc* tests. Significant differences were considered when *p < 0.05; **p < 0.01; ***p < 0.001. Error bars indicate the standard deviation or standard error of the mean for the technical replicates, as indicated in the figure legends. All statistical analyses were performed using R/Bioconductor (version 2.15.2).

### RNA-sequencing

RTT NPCs were transfected with LTV-control or LTV-miR106sp and differentiated into neurons. RNA was isolated with the RNeasy Mini Kit (Qiagen) at day 28 post-differentiation. RNA-seq was performed by Novogene Genome Sequencing Company using the Illumina Novoseq platform with a paired-end 150 bp sequencing strategy. Raw FASTQ files were trimmed using TRimmomatic (Galaxy version 23.0.rc1^86^). Reads were mapped to the human genome assembly GRCh38/hg38 using HiSAT2^87^. All sequencing data can be accessed via the Gene Expression Omnibus.

### PARIS2 assay

The PARIS assay was performed with modifications as described previously^28^. Briefly, control and miR106sp-treated H4SV cells were crosslinked with AMT (Sigma-Aldrich, A4330). The cross-linked RNA was extracted and fragmented using ShortCut RNase III (NEB, m0245). The purified and concentrated RNA fragments were proximity ligated by T4 RNA ligase 1 (NEB, M0437M). Proximity-ligated RNA fragments were irradiated with UV_254_ for 30 min. After reverse crosslinking, RNA was purified with three volumes of ethanol and 1 μl GlycoBlue. Isolated RNA was reverse transcribed using Superscript II Reverse Transcriptase (Invitrogen). Quantitative real-time PCR was performed as described previously^17^ using primers listed in Supplementary Data III. qRT-PCR amplicons were sequenced, and the data were processed as described previously^28^. Briefly, sequencing data were mapped to the manually mm10 *Xist*-miR106a genome using the STAR program with pre-determined parameters. The primary mapping alignments were extracted and filtered to remove low-confidence segments and assembled into DGs.

### ChIRP-MS

The ChIRP-MS assay was performed with modifications as described previously^10^. Briefly, 20–30 15-cm dishes of H4SV cells were cross-linked in 3% formaldehyde for 30 mins, followed by 0.125 M glycine quenching for 5 mins. The cross-linked cell lysate was sonicated, and protein-RNA complexes were pulled down using *Xist*-specific biotinylated probes^10^. Extracted *Xist*-bound protein complexes were size-separated in bis-tris SDS-PAGE gels for MS analysis.

The SDS-PAGE gel slices were cut into 1 mm^2^ cubes with a fresh razor blade and transferred to a clean microcentrifuge tube. The gel pieces were washed twice with 50% Acetonitrile (ACN) and 50 mM Ammonium Bicarbonate pH 8 (ABC) for 15 min with shaking. The gel pieces were dehydrated with 100% ACN for 5 min with shaking. Then the solvent was removed, and the gel pieces were allowed to air dry for 20 min. 10 mM Tris(2-carboxyethyl) phosphine, hydrochloride (TCEP) and 40 mM Chloroacetamide (CAA) were added to the dry gel pieces and incubated at 70 °C for 5 min. The gel pieces were rewashed with 50% ACN and 50% 50 mM ABC for 15 min with shaking. The gel pieces were rehydrated in 50 mM ABC, and one μg Trypsin/ Lys C (1:50) was added and incubated for 1 h at room temperature, then 50 mM HEPES pH 8.0 solution was added to cover the pieces. The samples were allowed to incubate overnight at 37 °C. Peptides were extracted from gel pieces with 25% ACN and 50 mM Ammonium Bicarbonate pH 8.0, then again with 100% ACN for 5 min with shaking. Samples were filtered through a 022-μm PVDF spin column (Millipore). Peptides were dried back in a Speedvac to 30 μl and acidified with two μl formic acid (NEAT).

The extracted peptides were run on electrospray ionization (ESI) using a Fusion Lumos Tribrid Orbitrap Mass Spectrometer (Thermo Fisher Scientific) coupled to a capillary nanoLC-MS (Thermo Fisher Easy-nLC1200) liquid chromatography with a FLEX ion source (Thermo Fisher). Chromatography of the peptides used a 25 cm reversed-phase column and a 10 cm precolumn fabricated in-house (75-μm inner diameter, packed with ReproSil-Gold C18-1.9-μm resin (Dr. Maisch GmbH)) that was equipped with a laser-pulled nanoelectrospray emitter tip (2 μm). The precolumn had 3.0 μm packing (Dr. Maisch GmbH) and a Kasil frit. Peptides were eluted at a 250 nl/min flow rate using a linear gradient of 2–40% buffer B in 70 min (buffer B: 0.05% formic acid and 95% acetonitrile in water). Data were acquired in Orbi-trap mode. Instrument method parameters were MS1 resolution, 120,000 at 200 m/z: scan range, 350 − 1600 m/z. The top 20 most abundant ions were subjected to higher energy collision-induced dissociation (HCD) with a normalized collision energy of 35%, activation q 0.25, and precursor isolation width 2 m/z. Dynamic exclusion was enabled with a repeat count of 1, a repeat duration of 30 s, and an exclusion duration of 20 s. Raw files were analyzed using PEAKS (Bioinformatics Solution Inc.). Peptide and protein hits were filtered using a 5% false discovery rate (FDR) with a minimum of 2 unique peptides per protein. The data was searched using PEAKS studio 12.0 software (build 20240723) and the full Mus musculus (Mouse) database with the following parameters: Semi-specific tryptic rules, peptide length range 6–45, carbamidomethylation (+57.02) as a fixed modification on cysteine and oxidation of methionine (+15.99) as a variable modification, five missed cleavages and four modifications per peptide, parent mass error tolerance of 15.0 ppm and 0.5 Da for fragment mass error tolerance. Label-free quantitation (LFQ) was performed using the PEAKS studio 12.0 software and the LFQ quantitation module with default parameters. The total ion current (TIC) was used for all normalization, including technical replicates. Potential MS artifacts were first filtered by removing low-confidence protein hits with fewer than seven peptides and selected for nuclear localization (GO terms). A stringent cut-off of significance score >15 and log_2_(fold change) greater than two between groups is applied to eliminate RNA-independent background interactions.

The in-gel Trypsin/Lys C-digested peptides were ionized using a Fusion Lumos Tribrid Orbitrap Mass Spectrometer (Thermo Fisher Scientific) and analyzed by online capillary nanoLC-MS (Thermo Fisher Easy-nLC1200). Raw files were analyzed using PEAKS (Bioinformatics Solution Inc.). Peptide hits were filtered using a 5% false discovery rate (FDR), and label-free quantitation (LFQ) was performed using the PEAKS quantitation module with default parameters, along with the total ion current (TIC) used for all normalization, including technical replicates. Potential MS artifacts were first filtered by removing low-confidence protein hits with fewer than seven peptides and selected for nuclear localization (GO terms). A stringent cut-off of significance score >15 and log_2_(fold change) greater than two between groups is applied to eliminate RNA-independent background interactions. A complete list of enriched 62 proteins with log_2_(fold change) was reported in Supplementary Data II.

### Reporting summary

Further information on research design is available in the Nature Portfolio Reporting Summary linked to this article.

## Supplementary information

Supplementary Information

Description of Additional Supplementary Files

Supplementary Data 1

Supplementary Data 2

Supplementary Data 3

Supplementary Movie IA

Supplementary Movie IB

Supplementary Movie IC

Supplementary Movie IIA

Supplementary Movie IIB

Supplementary Movie IIC

Supplementary Movie III

Supplementary Movie IV

Reporting Summary

Transparent Peer Review file

## Source data

Source data

## Data Availability

The data generated in this study are provided in the Source data file. The transcriptomic datasets generated in this study are publicly available in the NCBI Gene Expression Omnibus under accession code GSE261876. The sequence data for the PARIS data generated in this study are publicly available in the Sequence Read Archive under accession code PRJNA1254773. The mass spectrometry proteomics data have been deposited in the ProteomeXchange Consortium via the PRIDE [1] partner repository with the dataset identifier PXD064749 and 10.6019/PXD064749. Source data are provided with this paper.
