# Supplementary material for: Targeting microRNA-dependent control of X chromosome inactivation improves the Rett Syndrome phenotype
Source: Nat Commun. 2025 Jul 4;16:6169. doi: 10.1038/s41467-025-61092-7 (PMC12227778; doi:10.1038/s41467-025-61092-7)
Supplement: Supplementary file 1 — Supplementary Information [file 41467_2025_61092_MOESM1_ESM.pdf]

Targeting microRNA-dependent control of X chromosome inactivation improves the Rett Syndrome phenotype.

Song Lou[1], Rachisan DJiake Tihagam[1,a], Urszula N. Wasko[2,a], Zaffar Equbal[2], Sanjay Venkatesan[2], Klaudia Braczyk[1], Piotr Przanowski[2], Bon Il Koo[1], Ilyas Saltani[2], Arjun Tushir Singh[1], Shibi Likhite[3], Samantha Powers[3], George M. P. R. Souza[4], Robert A Maxwell[5], Jun Yu[6], Lihua J. Zhu[6], Mark Beenhakker[4], Stephen B. G. Abbott[4], Zhipeng Lu[7], Michael R. Green[6], Kathrin C. Meyer[3,8], Jogender Tushir-Singh[1], and Sanchita Bhatnagar[1,9,*]

[1]Department of Medical Microbiology and Immunology, University of California Davis School of Medicine, Davis, CA 95616, USA.

[2]Department of Biochemistry and Molecular Genetics, University of Virginia School of Medicine, Charlottesville, VA 22908, USA.

[3]Center for Gene Therapy, Nationwide Children's Hospital, OH 43205, USA.

[4]Department of Pharmacology, University of Virginia School of Medicine, Charlottesville, VA 22908, USA.

[5]The Vincent J. Coates Proteomics/Mass Spectrometry Core Laboratory, University of California, Berkeley, CA, USA.

[6]Department of Molecular, Cell, and Cancer Biology, University of Massachusetts Medical School, Worcester, MA 01605, USA.

[7]University of Southern California Alfred E. Mann School of Pharmacy and Pharmaceutical Sciences, USC Norris Comprehensive Cancer Center, USC Eli and Edythe Broad CIRM Center for Regenerative Medicine and Stem Cell Research, Los Angeles, CA 90033, USA.

[8]Department of Pediatrics, The Ohio State University, Columbus, OH 43205, USA

[9]The M.I.N.D. Institute, University of California at Davis, Davis, CA, USA.

[a]Authors contributed equally to the manuscript.

[*]Lead Contact

[*]To whom correspondence should be addressed:

Sanchita Bhatnagar

Department of Medical Microbiology and Immunology, the University of California Davis School of Medicine, 1275 Health Sciences Drive, Tupper Hall 3134, Davis, CA 95616, USA. Tel: 530-754-3074. Email: sbbhatnagar@ucdavis.edu

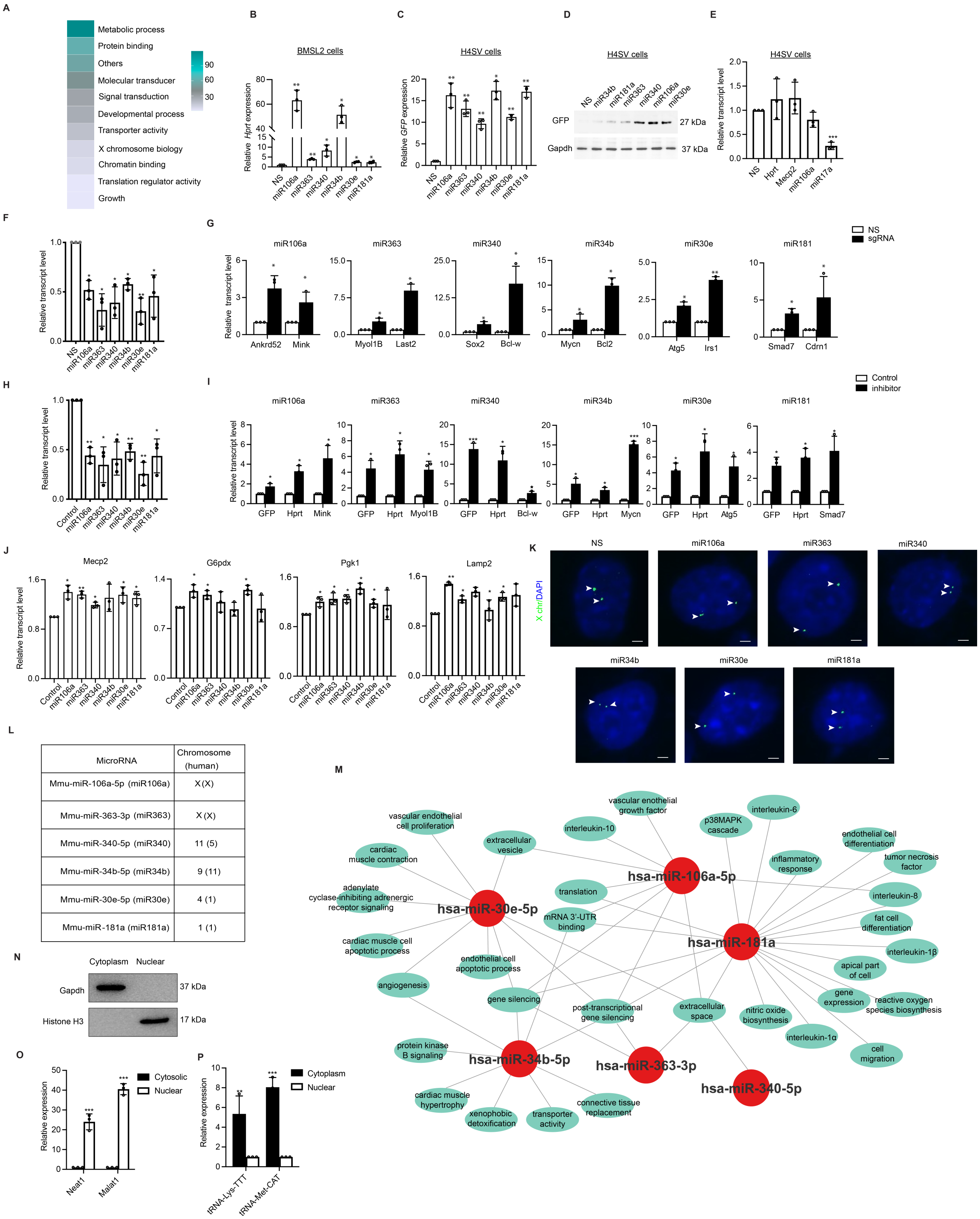

MicroRNA	Chromosome (human)
Mmu-miR-106a-5p (miR106a)	X (X)
Mmu-miR-363-3p (miR363)	X (X)
Mmu-miR-340-5p (miR340)	11 (5)
Mmu-miR-34b-5p (miR34b)	9 (11)
Mmu-miR-30e-5p (miR30e)	4 (1)
Mmu-miR-181a (miR181a)	1 (1)

Supplementary Figure 1. miRNAs are required for the silencing of X_i-linked genes. (A) Top 10 gene ontology (GO) processes for the protein-coding genes based on a consensus between biological replicates. (B) qRT-PCR analysis monitoring the expression of *Hprt* in the six-miRNA knockdown BMSL2 cells following HAT media selection. As a control, expression of *Hprt* in control cells is shown and was set to 1. (C-D) qRT-PCR (C) and western blot (D) analysis monitoring the expression of *GFP* in the six-miRNA knockdown H4SV cells following HAT media selection. (E) qRT-PCR analysis monitoring the expression of *Hprt*, *Mecp2*, miR106a, and miR17a in cells treated with miR17a inhibitor relative to control cells. (F-G) qRT-PCR analysis monitoring miRNA expression (F) and their target genes (G) in H4SV cells expressing NS or corresponding sgRNA. (H-I) qRT-PCR analysis monitoring the miRNAs (H) and the expression of *GFP*, *Hprt,* and miRNA target genes (I) in H4SV cells following treatment with the miRNA inhibitors. (J) qRT-PCR analysis monitoring *Mecp2*, *G6pdx*, *Pgk1* and *Lamp2* in the six-miRNA knockdown H4SV cells relative to control cells. (K) The representative images of DNA FISH monitoring X chromosome content in each of the six-miRNA knockdown H4SV cells. DAPI staining is shown in blue. (L) List of top-ranked miRNAs that derepressed X_i-linked genes. (M) Pathway functional network of the indicated miRNAs. (N) Western blot analysis of cytosolic and nuclear fractions from H4SV cells. (O-P) qRT-PCR analysis monitoring the expression of *Neat1* (O), *Malat1* (O)*, tRNA-Lys-TTT* (P), and *tRNA-Met-CAT* (P) in the nuclear and cytosolic fractions of H4SV cells. Data were analyzed from three experiments and expressed as mean ± SD (B, C, E-J, O-P). Unpaired, two-sided t-test with no multiple adjustments (B, C, E-J, O-P). (***$p < 0.001$; **$p < 0.01$; *$p < 0.05$). Scale bars: 5 µm (K).

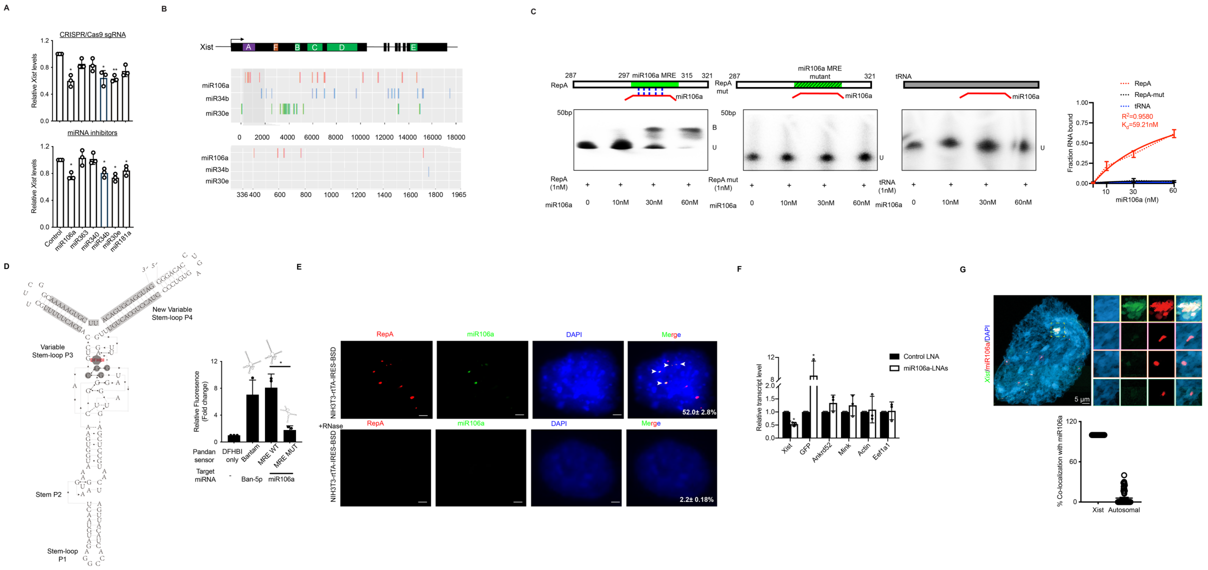
A
B
C
D
E
F
G
CRISPR/Cas9 sgRNA
miRNA inhibitors
Relative Xist levels
Control
miR106a
miR363
miR340
miR34b
miR30e
miR181a
Xist
miR106a
miR34b
miR30e
RepA
RepA mut
tRNA
miR106a MRE
miR106a MRE mutant
50bp
B
U
RepA (1nM)
RepA mut (1nM)
tRNA (1nM)
miR106a
0
10nM
30nM
60nM
Fraction RNA bound
RepA-mut
R^2=0.9580
K_d=59.21nM
miR106a (nM)
Variable Stem-loop P3
New Variable Stem-loop P4
Stem P2
Stem-loop P1
Relative Fluorescence (Fold change)
Pandan sensor
Target miRNA
DFHBI only
Bantam
MRE WT
MRE MUT
Ban-5p
NIH3T3-rtTA-IRES-BSD
+RNase
DAPI
Merge
52.0± 2.8%
2.2± 0.18%
Relative transcript level
Control LNA
miR106a-LNAs
GFP
Ankrd52
Mink
Actin
Eef1a1
Xist/miR106a/DAPI
5 µm
% Co-localization with miR106a
Autosomal

Supplementary Figure 2: miR106a binds to *Xist*. (A) qRT-PCR analysis monitoring the expression of *Xist* in the six-miRNA knockdown H4SV cells following treatment with either CRISPR/Cas9 sgRNA (*Top*) or synthetic inhibitors (*Bottom*). As a control, expression of *Xist* in control cells is shown, which was set to 1. (B) Schematic showing the predicted binding sites for the six-miRNA candidates in *Xist* (*Top*) and *Rep A* (*Bottom*). (C) The electrophoretic mobility shift assay of ^{32}P-labeled *RepA* with single-stranded miR106a at the indicated concentrations. A schematic of the *RepA* fragment with miR106a MRE is shown (*Top*). The miR106a-bound (B) and unbound (U) *RepA* are indicated for *RepA* and *RepA* mutant. tRNA is used as a control in the experiment. The experiment was performed thrice, and representative images from one experiment are shown. The binding curve of RepA fragments and miR106a from the three experiments is shown, and K_d is indicated. (D) Diagram showing the single-molecule version of RNA sensor as described in Methods (*Left*). Fluorescence intensity fold change of RNA sensor encoding wild type and mutant miR106a MRE in *RepA* (*Right*). Bantam-5p encoding RNA sensor is used as a positive control. (E) RNA-miRNA FISH monitoring *Xist* and miR106a localization in doxycycline-induced NIH3T3-rtTA-IRES-BSD nuclei. The doxycycline-induced NIH3T3-rtTA-IRES-BSD nuclei pre-treated with RNase were used as a negative control. The percent nuclei showing *Xist* and miR106a co-localization is indicated from three different experiments. (F) qRT-PCR monitoring *Xist* and X_i-linked genes in H4SV cells treated with miR106a-*RepA* specific locked nucleic acids (LNA). *Gapdh* is used as an endogenous control. (G) Confocal-airyscan sections of H4SV nuclei stained for *Xist* and miR106a in H4SV cells. Inserts are the enlargements of the *Xist* positive and negative regions (*Top*). The proportion of *Xist*-marked Xi's co-localizing with miR106a is quantitated (*Bottom*). Data were analyzed from three experiments and expressed as mean ± SD (A, C-G). Unpaired, two-sided t-test with no multiple adjustments (A, C-G). (***$p < 0.001$; **$p < 0.01$; *$p < 0.05$). Scale bars: 5 µm (E, G).

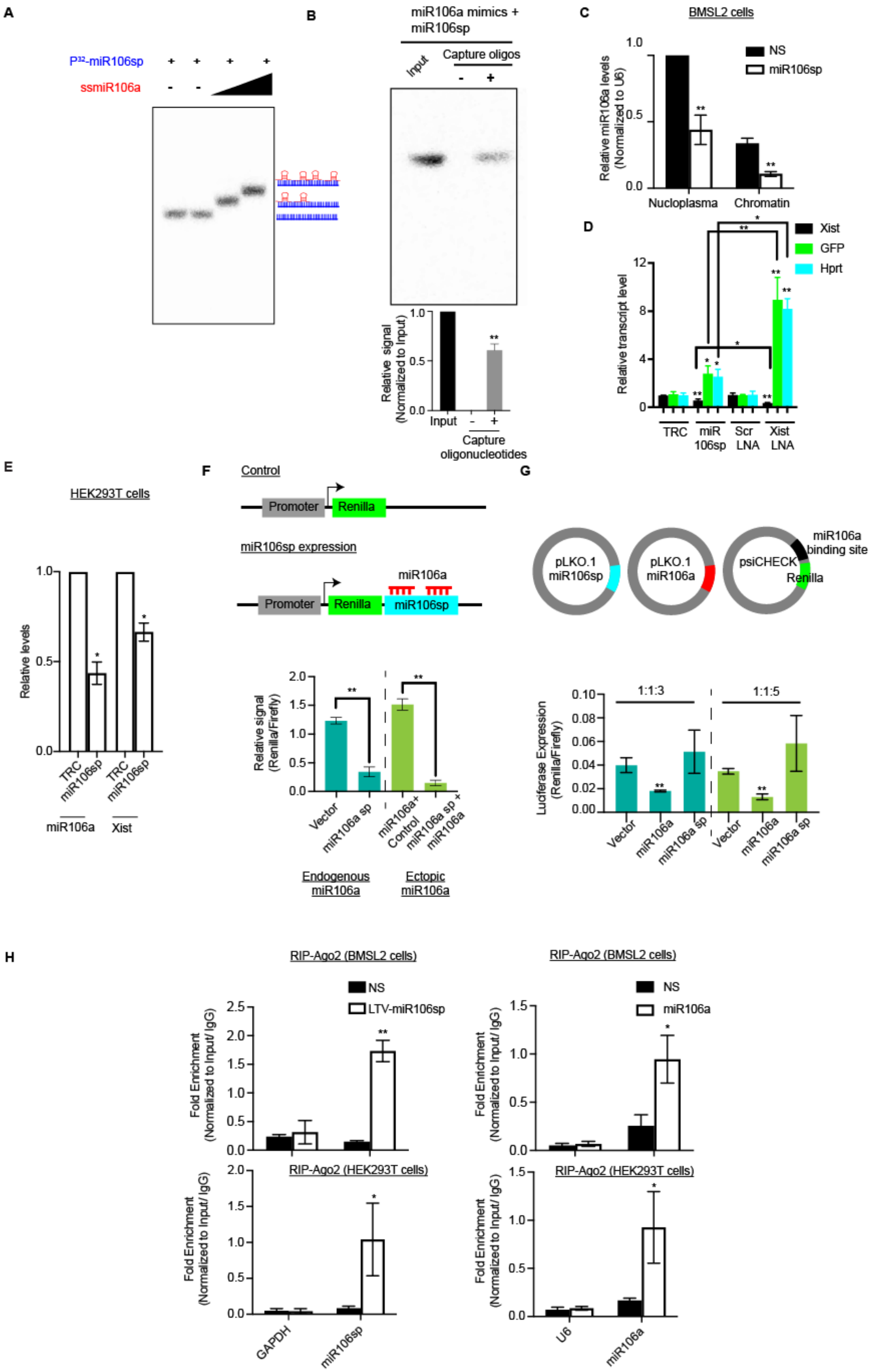

A
P32-miR106sp
ssmiR106a
B
miR106a mimics + miR106sp
Input
Capture oligos
Relative signal (Normalized to Input)
Capture oligonucleotides
C
BMSL2 cells
NS
miR106sp
Relative miR106a levels (Normalized to U6)
Nucleoplasma
Chromatin
D
Xist
GFP
Hprt
Relative transcript level
TRC
miR 106sp
Scr LNA
Xist LNA
E
HEK293T cells
Relative levels
TRC
miR106sp
miR106a
Xist
F
Control
Promoter
Renilla
miR106sp expression
miR106a
miR106sp
Relative signal (Renilla/Firefly)
Vector
miR106a sp
miR106a+ Control
miR106a sp × miR106a
Endogenous miR106a
Ectopic miR106a
G
pLKO.1 miR106sp
pLKO.1 miR106a
psiCHECK
miR106a binding site
Renilla
1:1:3
1:1:5
Luciferase Expression (Renilla/Firefly)
Vector
miR106a
miR106a sp
H
RIP-Ago2 (BMSL2 cells)
NS
LTV-miR106sp
miR106a
Fold Enrichment (Normalized to Input/ IgG)
RIP-Ago2 (HEK293T cells)
GAPDH
miR106sp
U6
miR106a

Supplementary Figure 3. miR106a-specific sponge sequence inhibits miR106a function. (A) Binding analysis of miR106a and *P^{32}-miR106sp* by northern blot. Lanes 1 and 2 correspond to the probe alone (miR106sp); lanes 3 and 4 correspond to the incubation of the probe with increasing concentrations of single-stranded miR106a (3 and 10 μM). (B) Competitive elution of *P^{32}-miR106sp* from the miR106a-*miR106sp* complex using capture oligonucleotides. The experiment was performed thrice, and a representative image from one experiment is shown (*Top*), and the results are quantified from three different experiments (*Bottom*). (C) qRT-PCR monitoring miR106a levels in the nucleoplasm and chromatin fraction of BMSL2 cells expressing NS or miR106sp. U6 is used as an endogenous control. (D) qRT-PCR monitoring *Xist* and X_i-linked genes in H4SV cells treated with either miR106sp or *Xist*-specific locked nucleic acids (LNA). *Gapdh* is used as an endogenous control. (E) qRT-PCR monitoring miR106a and *Xist* in HEK293T cells treated with miR106sp as described in the Methods. (F) *Top*, Schematic representation of miR106sp expressed from the T7 promoter in psiCHECK reporter plasmid. *Bottom*, Dual-Glo luciferase assays evaluating the *Renilla* suppression potentials of miR106sp in HEK293T cells expressing endogenous or ectopic miR106a. (G) *Top*, Schematic representation of psiCHECK reporter plasmid encoding perfect miR106a target sites and a pLKO.1 plasmid expressing miR106sp and miR106a. *Bottom*, Dual-Glo luciferase assays evaluating the *Renilla* levels in HEK293T cells expressing either miR106a or miR106sp. (H) RIP assay monitoring binding of Ago2 to miR106sp (*Left*) or miR106a (*Right*) in BMSL2 (*Top*) and HEK293T (*Bottom*) cells expressing NS or miR106sp. Data were analyzed from three experiments (C-H) and expressed as mean ± SD (B-H). Unpaired, two-sided t-test with no multiple adjustments (B-H). (***$p < 0.001$; **$p < 0.01$; *$p < 0.05$).

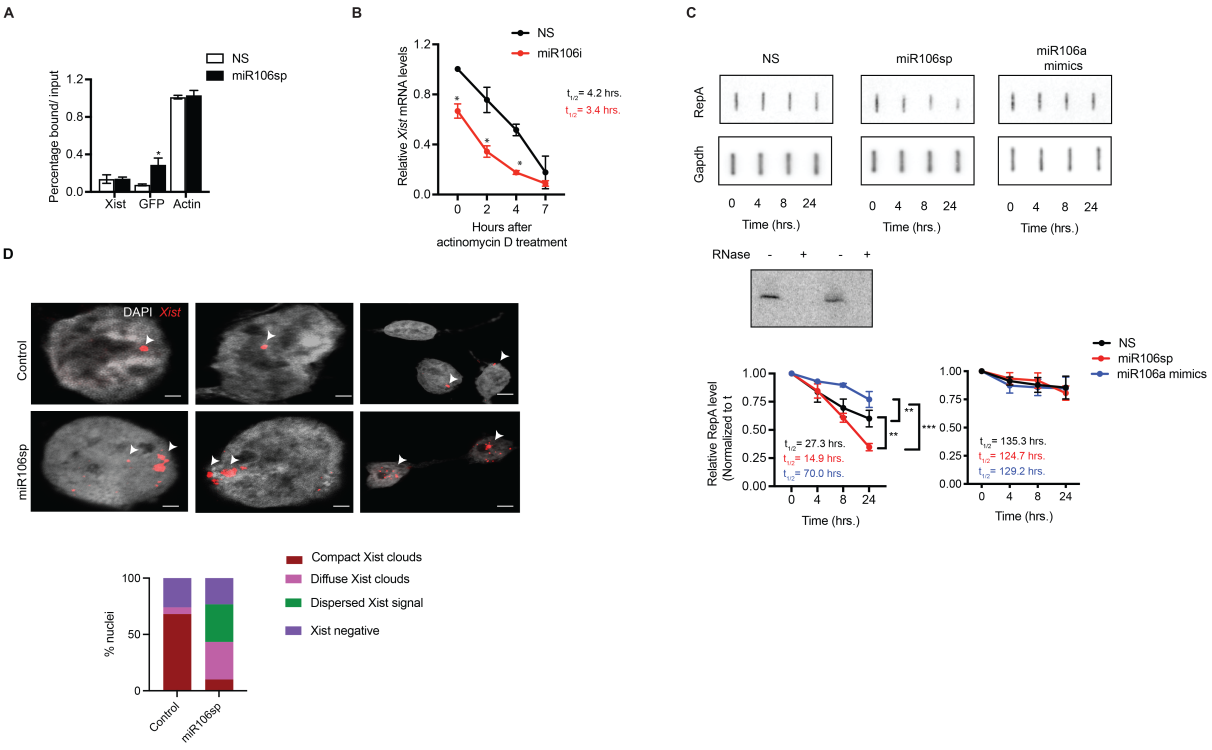
A
NS
miR106sp
Percentage bound/ input
1.2
0.8
0.4
0.0
Xist
GFP
Actin
B
NS
miR106i
Relative Xist mRNA levels
t1/2= 4.2 hrs.
t1/2= 3.4 hrs.
Hours after
actinomycin D treatment
C
NS
miR106sp
miR106a
mimics
RepA
Gapdh
0 4 8 24
Time (hrs.)
RNase - + - +
NS
miR106sp
miR106a mimics
Relative RepA level
(Normalized to t
t1/2= 27.3 hrs.
t1/2= 14.9 hrs.
t1/2= 70.0 hrs.
t1/2= 135.3 hrs.
t1/2= 124.7 hrs.
t1/2= 129.2 hrs.
Time (hrs.)
D
DAPI Xist
Control
miR106sp
Compact Xist clouds
Diffuse Xist clouds
Dispersed Xist signal
Xist negative
% nuclei
100
50
0
Control
miR106sp

Supplementary Figure 4. miR106a binding to *RepA* is required for the localization of *Xist* RNA to X_i. (A) ChIP analysis monitoring binding of RNA POLII to the Xist, GFP, and Actin promoter in H4SV cells expressing control or miR106sp. (B) qRT-PCR analysis monitoring *Xist* levels in H4SV cells treated with miR106sp inhibitor (miR106i) following actinomycin D. *Gapdh* mRNA was used as a normalization control. (C) Slot blot assay monitoring stability of *in vitro* synthesized capped *RepA* in a time-dependent manner. *In vitro*-synthesized *Gapdh* is used as an endogenous control. The representative images (*Top*) and the results quantified (*Bottom*) from three experiments are shown. *RepA* pre-treated with RNase was used as a negative control. (D) Confocal-airyscan sections of nuclei stained for *Xist* in control and miR106sp-treated MEFs. The proportion of *Xist*-positive cells is quantitated (*Bottom*). Data were analyzed from three experiments and expressed as mean ± SD (A-C). Unpaired, two-sided t-test with no multiple adjustments (A-C). (***$p < 0.001$; **$p < 0.01$; *$p < 0.05$). Scale bars: 2 µm (D, *Left*), 5 µm (D, *Right*).

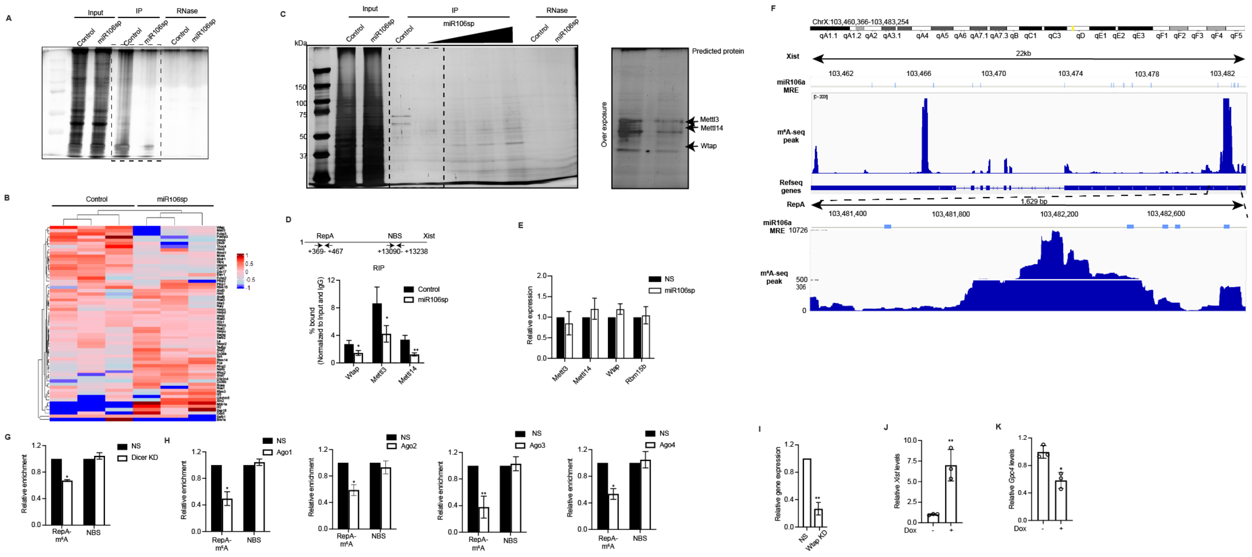
A
Input
IP
RNase
Control
miR106sp
B
Control
miR106sp
C
Input
IP
RNase
kDa
150
100
75
50
37
Over exposure
Predicted protein
Mettl3
Mettl14
Wtap
D
RepA
NBS
Xist
+369- +467
+13090- +13238
RIP
% bound
(Normalized to input and IgG)
Control
miR106sp
Wtap
Mettl3
Mettl14
E
Relative expression
NS
miR106sp
Mettl3
Mettl14
Wtap
Rbm15b
F
ChrX:103,460,366-103,483,254
Xist
22kb
miR106a MRE
m⁶A-seq peak
Refseq genes
RepA
1,629 bp
G
Relative enrichment
NS
Dicer KD
RepA-m⁶A
NBS
H
NS
Ago1
Ago2
Ago3
Ago4
I
Relative gene expression
NS
Wtap KD
J
Relative Xist levels
Dox
K
Relative Gpc4 levels

Supplementary Figure 5. miR106a depletion affects *Xist*-interacting proteins. (A) Silver-stained immunoblot of ChIRP-enriched proteins in control and miR106sp-treated cells. Input is 2% of the total protein, and the RNase control shows little background. (B) Heatmap of a subset of *Xist*-interacting proteins differentially pulled down in control and miR106a-depleted cells in the three replicates. Color bars indicate the abundance of peptides detected. (C) Silver-stained immunoblot of ChIRP-enriched proteins in control and miR106sp-treated cells. Input is 2% of the total protein, and the RNase control shows little background. The over-exposed lanes for ChIRP-enriched proteins in control and miR106sp-treated cells are shown (*Right*). (D) RIP assay monitoring Wtap, Mettl3, and Mettl14 interaction with *Xist* in H4SV cells expressing NS or miR106sp. (E) qRT-PCR monitoring Mettl3, Mettl14, Wtap, and Rbm15b in H4SV cells expressing NS or miR106sp. (F) Schematic illustrating the overlay of predicted miR106a MREs with *Xist* m^6A-seq data from[1] in the *Xist* (*Top*) and *RepA* region (*Bottom*). The miR106a MREs are indicated in *Red*. (G-H) m^6A-qRT-PCR monitoring m^6A levels on *Xist* in H4SV cells expressing NS, Dicer (G), or Ago (1-4; H) shRNA. (I) qRT-PCR analysis monitoring knockdown efficiency of Wtap in H4SV cells. (J-K) qRT-PCR analysis monitoring the expression of *Xist* (J) *and Gpc4* (K) in pSM33 Xist-$(BoxB)_3$ cells after 48 hours of induction with doxycycline. Data were analyzed from three experiments and expressed as mean ± SD (D-E, G-K). Unpaired, two-sided t-test with no multiple adjustments (D-E, G-K). (***p < 0.001; **p < 0.01; *p < 0.05).

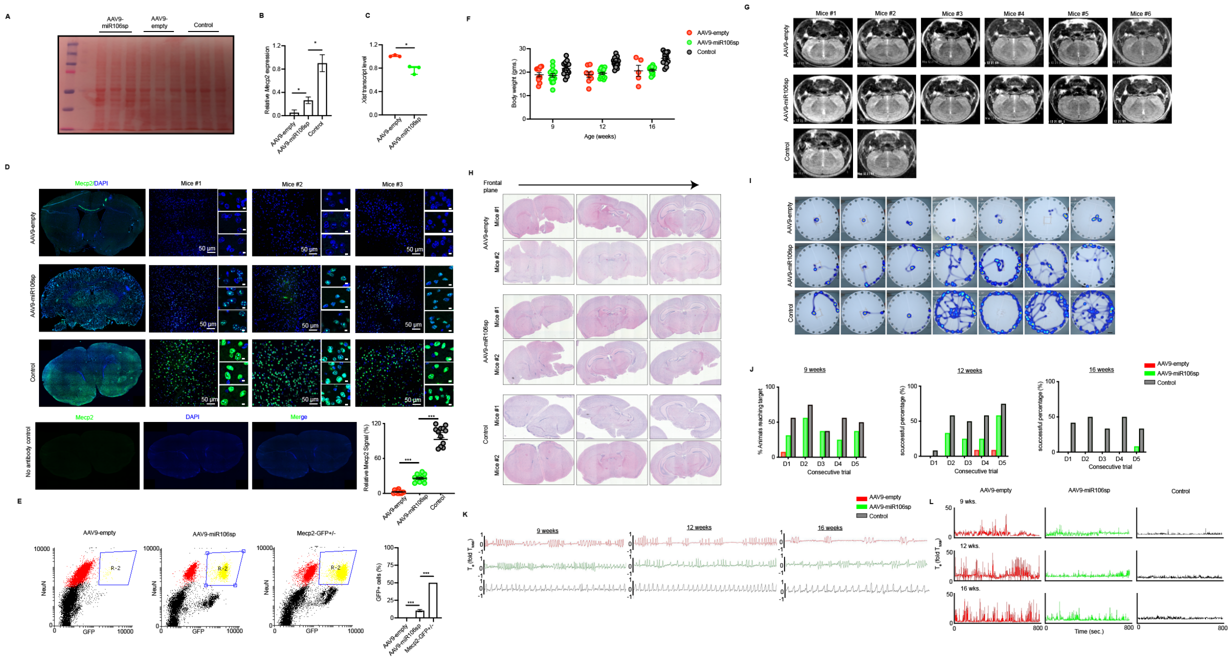

A
B
C
D
E
F
G
H
I
J
K
L

Supplementary Figure 6. AAV9-miR106sp-injected mice show improvement in *Mecp2* expression and behavioral phenotype. (A) Ponceau-stained immunoblot of Mecp2 levels in the whole-brain lysates derived from the AAV9-miR106sp and AAV9-empty-injected *Tsix-Mecp2* mice and control animals (n=3) at ~16 weeks. (B-C) qRT-PCR analysis monitoring *Mecp2* (B) and *Xist* (C) in the total RNA derived from the brain tissue of AAV9-miR106sp and AAV9-empty-injected *Tsix-Mecp2* mice (n=3 animals per group) at ~16 weeks. (D) Representative images and quantitation of Mecp2 (Green) staining in the coronal brain section from AAV9-empty-injected or AAV9-miR106sp-injected or control mice at ~16 weeks post-injection. DAPI stains the nucleus Blue. The whole brain image (n=1; *Left*) and the zoomed-in view (n=3, *Right*) are shown. Insets depict Mecp2 staining intensities. Representative image of no antibody control in the coronal brain section from control mice at ~16 weeks post-injection. The results are quantified from the three animals. (E) FACS analysis for *Mecp2-GFP* in NeuN+ nuclei isolated from the brain cells of AAV9-miR106sp and AAV9-empty-injected $Xist^{\Delta+/-}$*:Mecp2/Mecp2-GFP* mice. *Mecp2-Gfp+/-* animals are used as a control (n=3 animals per group). (F) Weights of AAV9-miR106sp (n=13) and AAV9-empty (n=14)-injected *Tsix-Mecp2* and control (n=14) mice are shown over time. The statistical analysis was done using Levene's test. (G) Representative MR images of the whole brain from age-matched AAV9-miR106sp (n=6) and AAV9-empty (n=6)-injected *Tsix-Mecp2* and control (n=2) mice at ~6 weeks. The control mice were used as a reference for the images and were excluded from the statistical analyses. (H) Representative H&E-stained sections of the whole brain (100X) from age-matched AAV9-miR106sp and AAV9-empty-injected *Tsix-Mecp2* and control mice sacrificed at ~16 weeks of age (n=2 animals per group). (I) Representative tracking data from individual Barnes maze trials for age-matched AAV9-miR106sp and AAV9-empty-injected *Tsix-Mecp2* and control mice. (n=12 animals per group). Scale bars: 10cm. (J) The percent success in the Barnes maze test for AAV9-empty-injected (n=13) or AAV9-miR106sp-injected (n=12) or control (n=12) mice at 9, 12, and 16 weeks. Data for the five consecutive days of evaluation is shown. The data were analyzed using an unpaired t-test. (K) Representative 10

sec. plethysmograph recordings of animals from each group at the indicated times. (L) Expiratory time (T_e; (fold T_{total})) for representative AAV9-empty or AAV9-miR106sp-injected or control mice as measured over 5 minutes. T_e is normalized to the mean breath duration during the 5-minute recording period for each mouse. Data were analyzed from three experiments and expressed as mean ± SD (B-E). Unpaired, two-sided t-test with no multiple adjustments (B-E). (***$p < 0.001$; **$p < 0.01$; *$p < 0.05$).

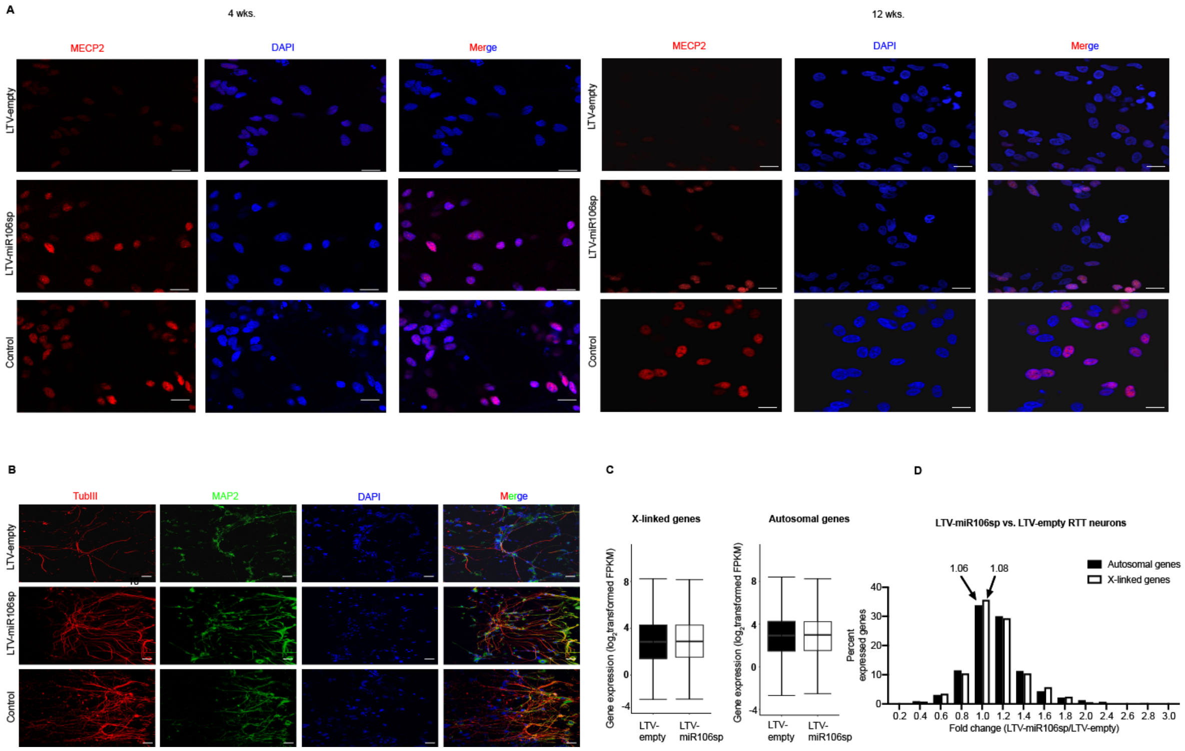
A
4 wks.
12 wks.
MECP2
DAPI
Merge
LTV-empty
LTV-miR106sp
Control
B
TubIII
MAP2
DAPI
Merge
C
X-linked genes
Autosomal genes
Gene expression (log2 transformed FPKM)
LTV-empty
LTV-miR106sp
D
LTV-miR106sp vs. LTV-empty RTT neurons
Autosomal genes
X-linked genes
1.06
1.08
Percent expressed genes
Fold change (LTV-miR106sp/LTV-empty)

Supplementary Figure 7. miR106a-mediated *MECP2* expression improves morphology and function in RTT neurons and figures related to the discussion. (A) The representative images show MECP2 (*Red*) staining in RTT neurons expressing empty or miR106sp and control neurons at ~4 and ~12 weeks post-differentiation. DAPI stains the nucleus Blue. (B) Representative images of MAP2 (*Green*) and TubIII (*Red*) staining in LTV-empty or LTV-miR106sp-infected RTT neurons and control neurons, ~12 weeks post-differentiation. DAPI stains the nucleus Blue. (C) Box plot of X-linked and autosomal gene expression (log_2-transformed FPKM) in RTT neurons expressing control or miR106sp (n = 2 per group). The boxed areas span the first to the third quartile, with the central line representing the median expression changes for each group. Outliers from the boxplots are not displayed. (D) Distribution of autosomal (*Black*) versus X-linked (*White*) fold changes in miR106sp-treated relative to empty-treated RTT neurons. Only genes with CPM ≥ 1 were considered to minimize the effects of noise. Fold changes are binned in steps of 0.2. Average fold changes of autosomal and X-linked genes are indicated. Scale bars: 20 μm (A), 40 μm (B).

Supplementary References

1. Coker, H. *et al.* The role of the Xist 5' m6A region and RBM15 in X chromosome inactivation. *Wellcome Open Res* **5**, 31 (2020).
