# Supplementary material for: Targeting microRNA-dependent control of X chromosome inactivation improves the Rett Syndrome phenotype
Source: Nat Commun. 2025 Jul 4;16:6169. doi: 10.1038/s41467-025-61092-7 (PMC12227778; doi:10.1038/s41467-025-61092-7)
Supplement: Supplementary file 2 — Description of Additional Supplementary Files [file 41467_2025_61092_MOESM2_ESM.pdf]

Description of Additional Supplementary Files

File Name: Supplementary Movie 1

Description: Phenotype of AAV9-empty-injected, AAV9- miR106sp-injected and control mice at ~12 weeks, showing smaller size, strong visible tremor, and reduced movement (video at 1x speed).

File Name: Supplementary Movie 2

Description: The comparison of hind-limb clasping phenotype in AAV9-empty-injected (A), AAV9-miR106sp-injected (B), and control (C) mice at ~11 weeks (video at 1x speed).

File Name: Supplementary Movie 3

Description: Open-field test with AAV9-empty-injected, AAV9- miR106sp-injected, and control mice at ~12 weeks, showing reduced movement and exploratory behavior (video at 1x speed).

File Name: Supplementary Movie 4

Description: Rotarod with AAV9-empty-injected, AAV9- miR106sp-injected and control mice at ~12 weeks, from left to right: AAV9- empty, AAV9-miR106sp, control that shows the variability in the performance of animals (video at 1x speed).

File Name: Supplementary Data 1

Description: List of protein-coding XCIFs identified through loss-of-function CRISPR/Cas9 screen. A list of genes enriched in each Gene ontology term and X chromosome biology is included.

File Name: Supplementary Data 2

Description: List of Xistinteracting proteins detected by ChIRP-MS.

File Name: Supplementary Data 3

Description: List of primers, sgRNA, probes, miRNA probes, RNA sensor, capture oligonucleotides, and shRNA used for qRT-PCR, ChIP, m6A-qRT-PCR, EMSA, RIP, and RNA sensor assay.
